# Sterol metabolism and protein metabolism are differentially correlated with sarcopenia in Asian Chinese men and women

**DOI:** 10.1111/cpr.12989

**Published:** 2021-02-20

**Authors:** Chun‐Wei Li, Kang Yu, Ng Shyh‐Chang, Guo‐Xun Li, Song‐Lin Yu, Hui‐Jun Liu, Bo Yang, Zi‐Yao Li, Yong‐Jie Zhao, Long‐Yu Xu, Jing Xu, Ling‐Juan Jiang, Rong‐Ji Liu, Xin‐Yuan Zhang, Shao‐Fei Li, Xiao‐Wei Zhang, Hai‐Yan Xie, Kang Li, Yi‐Xiang Zhan, Min Cui, Hang‐Bo Tao, Yao Li, Gao‐Shan Liu, Ke‐Min Ni, Dong‐Jing Li

**Affiliations:** ^1^ Department of Clinical Nutrition and Department of Health Medicine Peking Union Medical College Hospital Chinese Academy of Medical Sciences (CAMS) and Peking Union Medical College (PUMC) Beijing China; ^2^ Institute for Stem Cell and Regeneration State Key Laboratory of Stem Cell and Reproductive Biology Institute of Zoology Chinese Academy of Sciences (CAS) Beijing China; ^3^ University of Chinese Academy of Sciences Beijing China; ^4^ Department of Colorectal Surgery & Clinical Nutrition Tianjin Union Medical Center The Affiliated Hospital of Nankai University Tianjin China; ^5^ Department of Clinical Laboratory Peking Union Medical College Hospital Chinese Academy of Medical Sciences (CAMS) and Peking Union Medical College (PUMC) Beijing China; ^6^ Department of Clinical Nutrition Dongzhimen Hospital Beijing University of Traditional Chinese Medicine Beijing China; ^7^ Department of Orthopaedic Peking Union Medical College Hospital Chinese Academy of Medical Sciences (CAMS) and Peking Union Medical College (PUMC) Beijing China; ^8^ School of Medicine Nankai University Tianjin China; ^9^ Department of General Surgery Tianjin Union Medical Center The Affiliated Hospital of Nankai University Tianjin China; ^10^ Department of Sport Physiology Peking Union Medical College Hospital Chinese Academy of Medical Sciences (CAMS) and Peking Union Medical College (PUMC) Beijing China; ^11^ Department of Medical Research Center Peking Union Medical College Hospital Chinese Academy of Medical Sciences (CAMS) and Peking Union Medical College (PUMC) Beijing China; ^12^ Department of Pharmacy Peking Union Medical College Hospital Chinese Academy of Medical Sciences (CAMS) and Peking Union Medical College (PUMC) Beijing China; ^13^ Department of Stomatology Peking Union Medical College Hospital Chinese Academy of Medical Sciences (CAMS) and Peking Union Medical College (PUMC) Beijing China; ^14^ Department of Clinical Nutrition Hebei General Hospital Shijiazhuang China; ^15^ Department of Health Care and Department of Health Medicine Peking Union Medical College Hospital Chinese Academy of Medical Sciences (CAMS) and Peking Union Medical College (PUMC) Beijing China; ^16^ Department of Vascular Surgery Peking Union Medical College Hospital Chinese Academy of Medical Sciences (CAMS) and Peking Union Medical College (PUMC) Beijing China; ^17^ Department of Health Education Shijingshan Center for Disease Prevention and Control Beijing China

**Keywords:** BMI, hand grip strength, predictive model, sarcopenia, testosterone, vitamin D

## Abstract

**Objectives:**

Our aim was to investigate the prevalence and predictive variables of sarcopenia.

**Methods:**

We recruited participants from the Peking Union Medical College Hospital Multicenter Prospective Longitudinal Sarcopenia Study (PPLSS). Muscle mass was quantified using bioimpedance, and muscle function was quantified using grip strength and gait speed. Logistic regression revealed the relationships between sarcopenia and nutritional, lifestyle, disease, psychosocial and physical variables.

**Results:**

The prevalence of sarcopenia and sarcopenic obesity was 9.2%‐16.2% and 0.26%‐9.1%, respectively. Old age, single status, undernourishment, higher income, smoking, low physical activity, poor appetite and low protein diets were significantly associated with sarcopenia. Multiple logistic regression analysis showed that age was a risk factor for all stages of sarcopenia, and participants above 80 years were greater than fivefold more susceptible to sarcopenia, while lower physical activity was an independent risk factor. The optimal cut‐off value for age was 71 years, which departs from the commonly accepted cut‐off of 60 years. Female participants were greater than twofold less susceptible to sarcopenia than male participants. The sterol derivative 25‐hydroxyvitamin D was associated with fourfold lower odds of sarcopenia in male participants. Several protein intake variables were also correlated with sarcopenia. Based on these parameters, we defined a highly predictive index for sarcopenia.

**Conclusions:**

Our findings support a predictive index of sarcopenia, which agglomerates the complex influences that sterol metabolism and nutrition exert on male vs female participants.

## Graphical abstract

Dysregulated circadian clock genes were associated with glioma grades and the IDH status. Prognostic model suggests circadian clock genes affect glioma progression. The GO and GSEA enrichment analysis suggested dysregulated circadian clock genes can affect glioma through interfering cell cycle and influencing immunocytes infiltration.

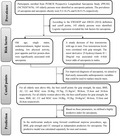

## 1 INTRODUCTION

Rapidly ageing populations around the world are experiencing an increase in muscle wasting syndromes. Sarcopenia is a progressive skeletal muscle wasting disorder that is associated with an increased likelihood of adverse outcomes, including falls, fractures, physical disability and mortality.^1^ Sarcopenia has been formally recognized as a muscle disease with an ICD‐10‐MC Diagnosis Code in some countries.^2^

In 1998, following the recommendation by Baumgartner et al,^3^ sarcopenia was defined with a cut‐off of a skeletal muscle index (SMI; appendicular skeletal muscle mass [ASM]/height^2^) that is more than two standard deviations (SD) below the mean for young and healthy adults. Subsequently, several regions including Europe, USA and Asia incorporated decreased physical performance as a diagnostic criterion.^4^ In 2010, the European Working Group on Sarcopenia in Older People (EWGSOP) published a sarcopenia definition that is now used worldwide.^5^ In 2014, the Asian Working Group on Sarcopenia (AWGS) further developed the EWGSOP‐based consensus by specifying cut‐off points for the diagnostic variables in Asians.^6^ Based on these criteria, the prevalence of sarcopenia was estimated to be 11.3% in women and 9.7% in men.^7^ Despite these efforts, reports on the prevalence of sarcopenia continue to vary widely between studies (10%‐50%), and they are difficult to compare because of the wide variance depending on the country of origin, the methods used and the diagnostic criteria.^8^

Several factors can influence muscle mass and strength, including muscular disuse and age‐related alterations in sex hormones, protein synthesis, proteolysis, neuromuscular integrity, endocrine function, nutritional balance and intramuscular fat content.^9^ Moreover, few studies have systematically surveyed the interactions between sarcopenia and all nutrient groups holistically, and even fewer studies focus on old adults.^10^ Sarcopenia has become the focus of intense research aiming to translate current knowledge about its pathophysiology into improved diagnosis and treatment, with particular interest in the development of biomarkers, nutritional interventions and drugs to become part of routine.^11^ Designing effective preventive strategies that people can apply during their lifetime is of primary concern. Hence, there is an urgent need to collect and report comprehensive data according to the best consensus criteria, to determine the cut‐off points for the appropriate diagnostic variables in Asian Chinese.

To address these limitations, our first aim was to determine the prevalence of sarcopenia in Asian Chinese male and female, using different established diagnostic criteria for skeletal muscle mass, namely EWGSOP and AWGS. Secondly, this study aimed to evaluate the association(s) between sarcopenia and common chronic illnesses, lifestyle factors, psychosocial well‐being and dietary nutrition patterns (including protein intake and sterol metabolism), in order to identify risk factors comprehensively and unbiasedly for sarcopenia. Thirdly, this study aimed to ascertain whether anthropometric indicators, such as hand grip strength, calf circumference (CC), fat percentage and body mass index (BMI), can be used to predict sarcopenia in situations where expensive diagnosis equipment is unavailable.

## 2 MATERIALS AND METHODS

### 2.1 Participants

All participants of the study were selected from the Peking Union Medical College Hospital (PUMC Hospital) Multicenter Prospective Longitudinal Sarcopenia Study (PPLSS), an ongoing nation‐wide interdisciplinary cross‐sectional and intervention cohort study, to evaluate changes in muscle mass, muscle strength and clinical outcomes among sarcopenic elderly persons in China. The PPLSS protocol was approved by the Human Ethics Committee of the PUMC Hospital (no. HS889). This trial was registered at clinicaltrials.gov as NCT02873676.

The population of the cross‐sectional study was recruited according to our PPLSS selected criteria.^12^ Data from the young adults aged 18‐44 years were used as reference data to define cut‐off values for normal skeletal muscle mass in this study.

### 2.2 Diagnostic measures for sarcopenia according to different consensus panels

According to the EWGSOP (2010) definition, sarcopenia was defined as participants with reduced muscle mass (SMI) and either low muscle strength (reflected by grip strength) or low physical performance (walking speed).^5^ However, in its 2019 definition, EWGSOP2 used low muscle strength as the primary parameter of sarcopenia.^1^ In this study, we used three methods to define sarcopenia, to compare the existing definitions with epidemiological data.^1^, ^3^, ^5^, ^6^ (a) Based on low muscle mass alone, sarcopenia was defined as the normal mean skeletal muscle mass below two or more SD for a younger reference group.^3^ (b) The EWGSOP (2010) and AWGS (2014) criteria defined the cut‐off points for SMI as <7.0 kg/m^2^ for men and <5.7 kg/m^2^ for women, the cut‐off points for low grip strength were <26 kg for men and <18 kg for women, and the cut‐off point for walking speed was ≤0.8 m/s.^6^ (c) According to the EWGSOP2 (2019) algorithm^1^, the cut‐off points for SMI were <7.0 kg/m^2^ for men and <5.5 kg/m^2^ for women, the cut‐off points for low grip strength were <27 kg for men and <16 kg for women, and the cut‐off point for gait speed ≤0.8 m/s. Pre‐sarcopenia was defined as low muscle mass,^5^ probable sarcopenia was defined as low muscle strength,^1^ and severe sarcopenia was defined as the presence of reduced muscle mass, strength and performance.^1^, ^5^

Muscle mass was measured by using a segmental multifrequency bioelectrical impedance analysis (M‐BIA) instrument that operated at frequencies of 1, 5, 50, 250, 500 and 1000 kHz (H‐Key350, Beijing Seehigher Technology Co., Ltd). Hand grip strength was measured by using an electronic hand dynamometer (CAMRY MODEL EH101, HaNDCReW). Physical function was assessed by the average walking speed over a 4‐m distance.^5^ The details of muscle mass and function measure were referenced as our previous study method part.^12^

The abdominal circumference (AC) was measured midway between the lateral lower rib margin and the superior anterior iliac crest at the end of a gentle expiration phase. CC was measured on the left leg in a seated position with the knee and ankle at right angles, feet resting on the floor. Mid‐upper arm circumference (MAC) was measured with anon‐stretchable measuring tape at a point equidistant between the acromion process of the left scapula and the olecranon process of the left ulna.

### 2.3 Data collection

Face‐to‐face interviews were conducted to complete a standardized, structured questionnaire to obtain information. The questionnaire used in the cross‐sectional study was developed specifically based on the Korea National Health and Nutrition Examination Survey (KNHANES)^13^ and combined with multidisciplinary expert discussion. The reliability, validity and acceptability of the questionnaire were analysed by a pilot study. The alpha coefficient was 0.6, the recovery was 96%, and the response rate was 95%. The time taken to complete the data collection ranged from 18.0 to 29.0 minutes depending on the participant's capacity to complete measurements, with an average of 15.0 ± 7.0 minutes across all subjects.

Demographic characteristics and lifestyle data were ascertained by an interviewer who administered the questionnaire at baseline. Occupations were classified into several major categories according to labour intensity and level of education. We defined participants to have a smoking habit if they had smoked more than 100 cigarettes and still smoked one pack (20 cigarettes) at least per month for more than 6 months. Alcohol intake was assessed by asking participants whether they were non‐drinkers, drank once a month, drank once a week and drank every day. The International Physical Activity Questionnaire (IPAQ) was used to evaluate the level of physical activity for all participants.^14^ The medical history, including the presence of diabetes, hypertension, hyperlipidaemia, was assessed by referring to the self‐reported physician's diagnosis. Activities of daily living were assessed using the Barthel index,^15^ and nutritional status was evaluated using the Mini‐Nutritional Assessment (MNA).^16^ Information on quality of life was obtained using the 5‐dimensional EuroQol (EQ‐5D).^17^ A trained interviewer asked each participant to report the frequency and the usual amount of consumption of each food item over the past year.

### 2.4 Vitamin D and testosterone measurements

Serum levels of 25‐hydroxyvitamin D (25OHD, including 25OHD_2_ and 25OHD_3_) and testosterone were measured at the Department of Clinical Laboratory (PUMC Hospital, China). The level of serum 25OHD was measured using liquid chromatography‐tandem mass spectrometry (LC‐MS/MS) system according to the previous reports.^18^ Total testosterone and sex hormone binding globulin (SHBG) levels were measured using an automated chemiluminescence immunoassay analyser (Beckman Coulter UniCel DXI 800, Beckman Coulter) using the corresponding reagents, calibration materials and quality control materials. The level of albumin (ALB) was measured using an automated chemistry analyser (Beckman Coulter AU5800, Beckman Coulter).

### 2.5 Data analysis

Data were analysed by using the statistical software EPIDATA 3.0. Analyses were performed by using SAS21.0.1 (SAS Institute). Continuous variables were summarized as means ± SD or medians (25th, 75th percentiles), and categorical variables were summarized as counts and percentages. Prevalence was based on a proportion of cases of sarcopenia among total study population. Subgroup analyses were conducted on the prevalence of sarcopenia based on demographics, lifestyle factors, and functional and clinical variables. The comparisons between groups were analysed using the chi‐squared test, Fisher exact test and Mann‐Whitney U test, where appropriate. We performed analysis of covariance to verify interrelationships between reduced muscle mass and related changes in physical function, and analysis of associations between nutritional parameters and BMI and muscle strength using Spearman's rank correlation. Multiple comparisons were made by the Nemenyi test. Conditional forward stepwise multiple logistic regressions were used to analyse the factors associated with the risk components. Most of the variables were categorized into two levels based on the median, while levels were subdivided into three levels based on the upper and lower quartile, to obtain the appropriate likelihood statistical power. The highest level was regarded as the reference group. The models included demographic variables, lifestyle variables, chronic conditions, anthropometric variables, dietary and nutritional variables. Non‐significant variables were omitted from models of the multiple logistic regression analyses to obtain the odds ratio (OR) and 95% confidence interval (CI). Receiver operating characteristic (ROC) analysis was performed to explore the cut‐off values of AC, MAC, CC, fat mass, hand grip strength and BMI for men and women, and to verify the predictive validity for sarcopenia. To eliminate the multicollinearity in establishing predictive model at the greatest extent, correlation analysis including variance inflation factor, tolerance, system of eigenvalues and Spearman's rank correlation was performed before the multivariable analysis. Conditional forward stepwise multiple logistic regressions were used again to establish the predictive model. Finally, Hosmer and Lemeshow tests were used to evaluate the exact of two predictive models. Differences were considered significant at *P* < .05.

For sample size calculations, we took previous AWGS‐based consensus sarcopenic prevalence estimates of 7.3% from a study of Chinese participants,^19^ with an error of 0.15P and an α level of 5% for the main variable, and it was estimated that 2260 adults would be required for this study. With allowance for a dropout for 20%, >2712 adults would meet the demand for sample size.

## 3 RESULTS

### 3.1 Participant inclusion criteria

**FIGURE 1:**
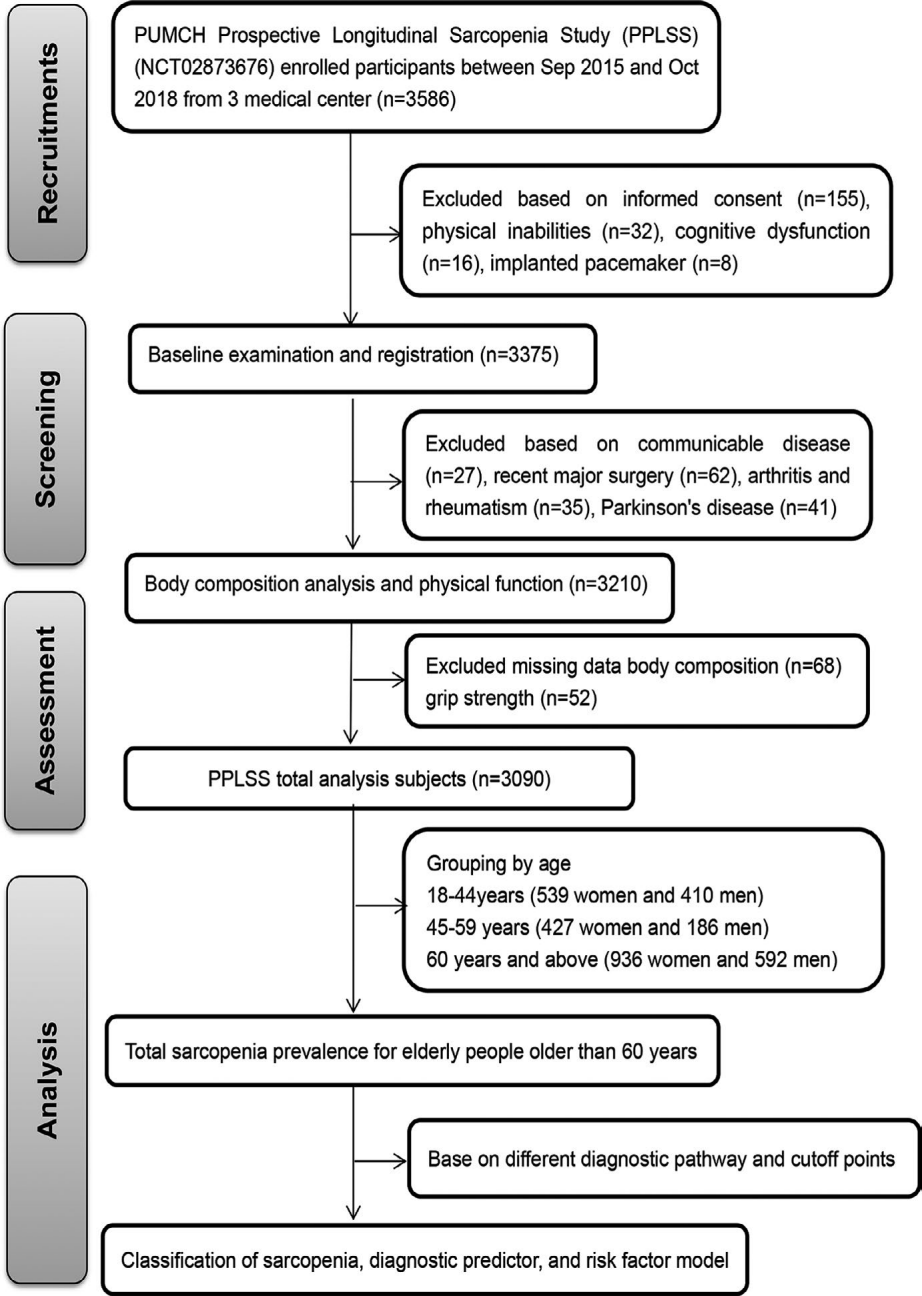
Flow chart of participants in the study. Samples for this study were enrolled from the Peking Union Medical College Hospital (PUMCH) Multicenter Prospective Longitudinal Sarcopenia Study (PPLSS). All samples selected were based on the relevant inclusion and exclusion criteria at every step

The flow chart for participant inclusion and exclusion in the study is shown in Figure 1. In total, 3586 participants were recruited during the data collection, of which 211 participants were considered ineligible to participate (73.5% subsequently refused to participate or failed to obtain guardian consent, 7.6% had cognitive dysfunction, and 3.8% had a pacemaker). Of the 3375 participants who finished the baseline examination and registration in PPLSS, 27 had communicable disease, 62 had received major surgery within the past 6 months, and 76 were diagnosed with Parkinson's disease, rheumatism or other diseases that might influence the results of the study. Of these, 3210 participants had finished body composition analysis and physical function evaluation. The records from 3090 participants were eventually considered complete, eligible and suitable for further analysis.

### 3.2 Baseline characteristics

The mean age of study population was 69.3 ± 7.7 years and ranged from 60 to 94 years. The BMI ranged between 15.1 and 43.0 kg/m^2^. Relative skeletal muscle mass index (RSMMI) ranged from 5.1 to 9.9 kg/m^2^ in male and from 2.5 to 8.5 kg/m^2^ in female. The hand grip strength ranged from 9.2 to 67.2 kg in male and from 5.1 to 56.4 kg in female. The walking speed was 0.95 ± 0.35 m/s. 97.3% of the participants were completely independent, 1.7% were slightly dependent, 0.1% were moderately dependent, and 0.7% were severely dependent. 47.6% of the participants had normal nutritional status, 49.6% were at risk of malnutrition, and 2.8% were malnourished.

### 3.3 Prevalence of sarcopenia and sarcopenic obesity

We considered three clinical definitions of sarcopenia in our study: (a) the Baumgartner definition, (b) the EWGSOP and AWGS (2014) cut‐off points and (c) the EWGSOP2 (2019) cut‐off points. According to the Baumgartner definition, sarcopenia is present in subjects whose muscle mass fall more than two SD below the young adults’ mean values (Table 1; 41.7 kg for male and 29.8 kg for female in our study population). Thus, 8.8% of our study population had sarcopenia, according to the early Baumgartner definition.

**TABLE 1:** Baseline characteristics in study population

	Young adults			Middle‐age adults			Elderly adults			*P* value^b^	*P* value^c^	*P* value^d^	*P* value^e^
	Male	Female	*P* value^a^	Male	Female	*P* value	Male	Female	*P* value				
N (%)	410 (43.2)	539 (56.8)		186 (30.3)	427 (69.7)		592 (38.74)	936 (61.26)					
Age (years)	27.7 ± 6.64	30.4 ± 6.43	<.001	53.3 ± 4.03	53.1 ± 3.88	.43	69.6 ± 7.82	69.1 ± 7.64	.15	<.001*			
Education level, n (%)													
Master or above	65 (15.9)	96 (17.8)	<.001	15 (8.1)	11 (2.6)	<.001	10 (1.7)	17 (1.8)	.001	<.001**	<.001	<.001	<.001
Bachelor or Junior college	188 (45.9)	379 (70.3)		48 (25.8)	168 (39.3)		136 (23.0)	152 (16.2)					
High or secondary school	127 (31.0)	41 (7.1)		64 (34.4)	159 (37.2)		114 (19.3)	210 (22.4)					
Junior middle school	28 (6.8)	17 (3.2)		50 (26.9)	74 (17.3)		224 (37.8)	300 (32.1)					
Primary school or lower	2 (0.5)	6 (1.1)		9 (4.8)	15 (3.5)		108 (18.2)	257 (27.5)					
Living situation, n (%)													
Living with three generations	82 (20.0)	174 (32.5)	<.001	22 (11.8)	36 (8.5)	.20	32(5.4)	52(5.6)	.032	<.001	<.001	<.001	<.001
Living with spouse and kids	54 (13.2)	130 (24.3)		74 (39.8)	152 (35.8)		133 (22.5)	210 (22.4)					
Living with parents	19 (4.6)	13 (2.4)		8 (4.3)	10(2.4)		16 (2.7)	51 (5.4)					
Living with spouse	36 (8.8)	219 (53.3)		71 (38.2)	195 (45.9)		314 (53.0)	451 (48.2)					
Alone	69 (12.9)	149 (27.9)		11 (5.9)	32 (7.5)		97 (16.4)	172 (18.4)					
Working type, n (%)													
Relative high‐intensity	198 (48.3)	8 (1.5)	<.001	1 (0.5)	2 (0.5)	1.0	12 (2.0)	11 (1.2)	.18	<.001	<.001	<.001	.054
Relative low‐intensity	212 (51.7)	531 (98.5)		185 (99.5)	425 (99.5)		580 (98.0)	925 (98.8)					
Income, n (%)													
More than $732.12	87 (21.2)	184 (34.1)	<.001	31 (16.7)	98 (23.0)	.039	86 (14.5)	76 (8.1)	<.001	< .001	<.001	<.001	<.001
$585.69‐$732.12	91 (22.2)	80 (14.8)		32 (17.2)	60 (14.1)		100 (16.9)	136 (14.5)					
$439.27‐$585.69	102 (24.9)	94 (17.4)		23 (12.4)	76 (17.8)		126 (21.3)	192 (20.5)					
$292.85‐$439.27	48 (11.7)	81 (15.0)		64 (34.4)	140 (32.8)		168 (28.4)	301 (32.2)					
Less than $292.85	82 (20.0)	100 (18.6)		36 (19.40)	53 (12.4)		112 (18.9)	231 (24.7)					
Marital status, n (%)													
Married	199 (48.5)	333 (61.8)	<.001	170 (91.4)	383 (89.7)	.72	495 (83.6)	673 (71.9)	<.001	< .001	<.001	<.001	<.001
Separated	207 (50.5)	199 (36.9)		3 (1.6)	10 (2.3)		13 (2.2)	28 (3.0)					
Divorced	4 (1.0)	3 (0.9)		5 (2.7)	18 (4.2)		7 (1.2)	22 (2.4)					
Widowed	0	2 (0.4)		8 (4.3)	16 (3.7)		76 (12.8)	213 (22.8)					
Smoking, n (%)													
Never	205 (50.0)	535 (99.3)	<.001	89 (47.8)	411 (96.3)	<.001	309 (55.6)	900 (96.2)	<.001	< .001	< .001	< .001	.006
Former	15 (3.7)	1 (0.2)		21 (11.3)	8 (1.9)		117 (19.8)	11 (1.2)					
Current	190 (46.3)	3 (0.6)		76 (40.9)	8 (1.9)		146 (24.7)	25 (2.7)					
Alcohol drinking, n (%)													
Never	190 (46.3)	462 (85.7)	<.001	80 (43.0)	362 (84.8)	<.001	375 (63.3)	875 (93.5)	<.001	<.001	<.001	<.001	<.001
Once or twice a month	74 (18.1)	53 (9.8)		23 (12.4)	29 (6.8)		38 (6.4)	26 (2.8)					
Once or twice a week	119 (29.0)	18 (3.3)		44 (23.7)	29 (6.8)		101 (17.1)	25 (2.7)					
Almost every day	27 (6.6)	6 (1.1)		39 (21.0)	7 (1.6)		79 (13.3)	10 (1.1)					
Comorbidities, n (%)													
Hypertension	6 (2.2)	9 (2.4)	.82	35 (36.1)	75 (24.1)	.02	173 (29.2)	328 (35.0)	.89	<.001	<.001	<.001	<.001
Hyperlipidemia	10 (3.6)	22 (6.0)	.17	22 (22.7)	84 (27.0)	.40	81 (13.7)	244 (26.1)	<.001	<.001	<.001	<.001	.15
Coronary heart disease	0	3 (0.8)	.26	14 (14.4)	21 (8.8)	.018	82 (13.9)	159 (17.0)	.92	<.001	<.001	<.001	<.001
Diabetes	1 (0.2)	7 (1.3)	.15	17 (9.1)	50 (11.7)	.35	98 (16.6)	176 (14.4)	.26	<.001	<.001	<.001	<.001
Osteoarthritis	2 (0.7)	10 (2.7)	.064	6 (6.2)	50 (16.1)	.013	40 (6.8)	176 (18.8)	<.001	<.001	<.001	<.001	.005
Osteoporosis	4 (1.0)	5 (0.9)	1.0	7 (3.8)	62 (14.5)	<.001	60 (11.1)	217 (23.2)	<.001	<.001	<.001	<.001	<.001
Fracture	18 (4.4)	13 (2.4)	.089	15 (8.1)	36 (8.4)	.88	36 (6.1)	121 (12.9)	<.001	<.001	<.001	<.001	.17
Respiratory disease	4 (1.4)	9 (2.4)	.37	4 (4.1)	17 (5.5)	.79	27 (4.6)	47 (5.0)	.68	<.001	.005	<.001	.27
Cancer	2 (0.50)	1 (0.2)	.58	7 (3.8)	13 (3.0)	.65	26 (4.4)	32 (3.4)	.33	<.001	<.001	<.001	.53
Digestive disease	9 (3.3)	24 (6.5)	.063	6 (6.2)	25 (8.0)	.55	35 (5.9)	84 (9.0)	.23	<.001	.099	<.001	.054
Renal dysfunction	1 (0.4)	1 (0.3)	1.0	4 (4.1)	8 (2.6)	.49	24 (4.1)	40 (4.3)	.58	<.001	<.001	<.001	.024
Hepatic dysfunction	11 (4.0)	4 (1.1)	.016	5 (5.2)	8 (2.6)	.20	11 (1.9)	33 (3.5)	.18	.17	.40	.063	.47
Nutrition status, n (%)													
Malnutrition risk	170 (41.6)	339 (63.0)	<.001	95 (51.1)	201 (47.6)	.045	226 (38.2)	413 (44.1)	.012	.021	.006	.08	.15
Malnutrition	6 (1.5)	11 (2.0)		0	8 (1.9)		26 (4.4)	22 (2.4)					
Appetite, n (%)													
Strong	165(40.2)	229(42.5)	.23	68(36.6)	143(33.5)	.32	189(31.9)	253(27.0)	.098	<.001	.01	<.001	.037
General	243(59.3)	304(56.4)		117(62.9)	275(64.4)		389(65.7)	665(71.0)					
Poor	1(0.2)	6(1.1)		1(0.5)	8(1.9)		13(2.2)	18(1.9)					
Fish frequency													
Almost everyday	28(6.8)	23(4.3)	.051	14(7.5)	25(5.9)	.042	24(4.1)	41(4.4)	.12	<.001	.013	<.001	.17
Less once every week	342(83.4)	444(82.4)		133(71.5)	343(80.5)		441(75.0)	737(78.9)					
None	38(9.3)	71(13.2)		39(21.0)	58(13.6)		123(20.9)	156(16.7)					
Poultry frequency													
Almost everyday	128(31.2)	104(19.3)	<.001	10(5.4)	33(7.7)	.55	62(10.5)	76(8.1)	.26	<.001	<.001	<.001	.14
Less once every week	264(64.4)	389(72.2)		145(78.0)	320(75.1)		435(74.0)	702(75.2)					
None	16(3.9)	45(8.4)		31(16.7)	73(13.1)		91(15.5)	156(16.7)					
Meat frequency													
Almost everyday	296(72.2)	287(53.3)	<.001	118(63.4)	206(48.4)	.003	297(50.5)	394(42.2)	.006	<.001	.003	<.001	.001
Less once every week	101(24.6)	225(41.7)		59(31.7)	187(43.9)		251(42.7)	465(49.8)					
None	11(2.7)	26(4.8)		9(4.8)	33(7.7)		40(6.8)	75(8.0)					
Milk frequency													
Almost everyday	122 (29.8)	198 (36.7)	.034	35 (18.8)	172 (40.4)	<.001	229 (38.9)	451 (48.3)	<.001	<.001	<.001	<.001	<.001
Almost every week	202 (49.3)	223 (41.4)		50 (26.9)	124 (29.1)		126 (21.4)	199 (21.3)					
None	84 (20.5)	117 (21.7)		101 (54.3)	130 (30.5)		233 (39.6)	284 (30.4)					
Dairy product													
Milk	253 (61.7)	253 (46.9)	<.001	62 (33.3)	210 (49.3)	<.001	295 (50.2)	493 (52.8)	<.001	<.001	<.001	<.001	<.001
Yogurt	66 (6.1)	153 (28.4)		20 (10.8)	74 (17.4)		49 (8.3)	141 (15.1)					
Cheese	15 (3.7)	34 (6.3)		11 (5.9)	21 (4.9)		20 (3.4)	33 (3.5)					
Powder	2 (0.5)	6 (1.1)		2 (1.1)	7 (1.6)		6 (1.0)	13 (1.4)					
None	72 (17.6)	92 (17.1)		91 (48.9)	114 (26.8)		218 (37.1)	254 (27.2)					
Bean frequency													
Almost everyday	198 (48.3)	192 (35.6)	<.001	73 (39.2)	150 (35.2)	.63	228 (38.8)	294 (31.5)	.013	.001	.11	< .001	.14
Less once every week	188 (45.9)	305 (56.6)		98 (52.7)	239 (56.1)		300 (51.0)	528 (56.5)					
None	22 (5.4)	41 (7.6)		15 (8.1)	37 (8.7)		60 (10.2)	112 (12.0)					
Nut frequency													
Almost everyday	32 (7.8)	90 (16.7)	<.001	30 (16.1)	135 (31.7)	<.001	134 (22.7)	324 (34.7)	<.001	<.001	<.001	<.001	<.001
Less once every week	198 (48.3)	291 (54.0)		83 (44.6)	165 (38.7)		171 (29.0)	291 (31.2)					
None	178 (43.4)	157 (29.1)		73 (39.2)	126 (29.6)		285 (48.3)	319 (34.2)					
Protein intake (g/day)													
Total protein	58.28 ± 21.98	46.58 ± 15.43	<.001	45.20 ± 18.00	45.73 ± 16.42	.61	46.13 ± 17.73	44.04 ± 14.27	.09	<.001	<.001	<.001	.51
Animal protein	32.01 ± 18.72	23.23 ± 12.36	<.001	20.60 ± 12.31	22.11 ± 10.97	.013	21.44 ± 11.89	21.05 ± 10.32	.99	<.001	<.001	<.001	.64
Vegetable protein	27.98 ± 12.14	23.67 ± 9.46	<.001	24.81 ± 10.96	23.85 ± 10.35	.44	24.73 ± 10.56	23.11 ± 8.45	.007	.001	.011	< .001	.84
Grain intake (g/day)													
≥300 g	122 (29.8)	63 (11.7)	<.001	56 (30.1)	54 (12.7)	<.001	133 (22.6)	132 (14.1)	<.001	.001	.28	<.001	.13
200‐300 g	121 (29.5)	154 (28.6)		54 (29.0)	152 (35.7)		221 (37.6)	338 (36.2)					
100‐200 g	130 (31.7)	207 (38.4)		49 (26.3)	160 (37.6)		172 (29.3)	361 (38.7)					
<100 g	35 (8.5)	114 (21.2)		27 (14.5)	60 (14.1)		62 (10.5)	102 (10.9)					
Fish intake (g/meal)													
≥150 g	110 (26.8)	102 (18.9)	.028	42 (22.6)	77 (18.1)	.097	80 (13.6)	116 (12.4)	.15	<.001	.33	<.001	.001
100‐150 g	103 (25.1)	139 (25.8)		38 (20.4)	112 (26.3)		122 (20.7)	226 (24.2)					
50‐100 g	111 (27.1)	169 (31.4)		50 (26.9)	136 (31.9)		193 (32.8)	328 (35.1)					
0 g	84 (20.5)	128 (23.8)		56 (30.1)	101 (23.7)		193 (32.8)	264 (28.3)					
Meat intake (g/meal)													
≥150 g	89 (21.7)	51 (9.5)	<.001	20 (10.8)	36 (8.5)	.43			.16	<.001	<.001	<.001	<.001
100‐150 g	126 (30.7)	145 (26.9)		38 (20.4)	87 (20.4)		172 (29.4)	233 (24.9)					
50‐100 g	137 (33.4)	194 (36.0)		74 (39.8)	153 (35.9)		201 (34.3)	328 (35.1)					
0 g	56 (13.7)	148 (27.5)		54 (29.0)	150 (35.2)		181 (30.9)	329 (35.2)					
Poultry intake (g/meal)													
≥150 g	102 (24.9)	68 (12.6)	<.001	24 (12.9)	41 (9.6)	.26	43 (7.3)	50 (5.4)	.34	<.001	<.001	<.001	.002
100‐150 g	129 (31.5)	163 (30.2)		46 (24.7)	101 (23.7)		135 (23.0)	223 (23.9)					
50‐100 g	130 (31.7)	192 (35.6)		56 (30.1)	161 (37.8)		247 (42.0)	379 (40.6)					
0 g	47 (11.5)	115 (21.3)		60 (32.3)	123 (28.9)		163 (27.7)	282 (30.2)					
Vegetable intake (g/d)													
≥500 g	87 (21.22	110 (20.41)	.008	64 (34.4)	127 (29.8)	.056	159 (27.0)	269 (28.8)	.25	<.001	<.001	<.001	.46
250‐500 g	177 (43.17)	204 (37.85)		65 (34.9)	183 (43.0)		263 (44.7)	391 (41.9)					
<250 g	115 (28.05)	202 (37.48)		48 (25.8)	109 (25.6)		149 (25.3)	256 (27.4)					
0 g	29 (7.07)	22 (4.08)		9 (4.8)	7 (1.6)		18 (3.1)	17 (1.8)					
Bean intake (g/meal)													
≥150g	24 (5.85)	39 (7.24)	.39	20 (10.8)	47 (11.0)	.74	43 (7.3)	54 (5.8)	.11	<.001	.02	.001	<.001
100‐150g	110 (26.83)	149 (27.64)		45 (24.2)	121 (28.4)		211 (35.9)	301 (32.3)					
50‐100g	142 (34.63)	202 (37.48)		70 (37.6)	149 (35.0)		197 (33.5)	367 (39.3)					
0g	132 (32.2)	148 (27.46)		51 (27.4)	109 (25.6)		137 (23.3)	211 (22.6)					
Nut intake (g/time)													
≥50g	75 (18.29)	68 (12.62)	<.001	35 (18.8)	65 (15.3)	.019	69 (11.7)	97 (10.4)	<.001	.001	.2	.006	.001
20‐50g	96 (23.41)	152 (28.2)		45 (24.2)	141 (33.1)		132 (22.4)	286 (30.6)					
<20g	58 (14.15)	162 (30.06)		32 (17.2)	94 (22.1)		100 (16.9)	232 (24.8)					
0g	178 (43.41)	156 (28.94)		74 (39.8)	126 (29.6)		289 (49.0)	319 (34.2)					
Salt intake (g/day)													
<3 g	28 (6.83)	52 (9.65)	<.001	16 (8.6)	41 (9.6)	.002	53 (9.0)	122 (13.1)	.012	<.001	<.001	<.001	<.001
3‐5 g	105 (25.61)	222 (41.19)		50 (26.9)	165 (38.7)		246 (41.7)	417 (44.6)					
5‐8 g	111 (27.07)	194 (35.99)		78 (41.9)	169 (39.7)		202 (34.2)	288 (30.8)					
>8 g	164 (40)	70 (12.99)		42 (22.6)	51 (12.0)		89 (15.1)	107 (11.5)					
Caffeine drinking													
Yes	223 (54.4)	314 (58.3)	.25	91 (48.9)	209 (49.1)	.98	331 (56.1)	638 (68.3)	<.001	<.001	.003	.001	<.001
No	185 (45.1)	224 (41.6)		95 (51.1)	217 (50.9)		259 (43.9)	296 (31.7)					
Protein distribution													
Three meals	271 (66.1)	214 (39.7)	<.001	76 (40.9)	181 (42.6)	.77	244 (41.4)	427 (45.7)	.024	.001	.002	.002	.32
Two meals	112 (27.3)	237 (44.0)		82 (44.1)	189 (44.5)		266 (45.2)	357 (38.2)					
One meal	25 (6.1)	87 (16.1)		28 (15.1)	55 (12.9)		79 (13.4)	150 (16.1)					
Supplements, n (%)													
Multivitamin and minerals	13 (3.2)	57 (10.6)	<.001	6 (3.2)	34 (8.0)	<.001	38(6.4)	89 (9.5)	.001	<.001	<.001	<.001	.48
Calcium and vitamin D	14 (3.1)	39 (7.2)		6 (3.2)	60 (14.1)		42 (7.1)	119 (12.7)					
Whey protein	5 (1.2)	8 (1.5)		2 (1.1)	10 (2.3)		9 (1.5)	18 (1.9)					
Fish oil	0	5 (0.9)		3 (1.6)	13 (3.0)		10 (1.7)	17 (1.8)					
Height (cm)	174.3 ± 5.45	161.9 ± 4.80	<.001	170.5 ± 6.00	160.0 ± 5.02	<.001	168.5 ± 6.49	157.3 ± 5.71	<.001	<.001	<.001	<.001	<.001
Weight (kg)	76.4 ± 13.29	58.5 ± 9.49	<.001	75.7 ± 10.99	63.5 ± 10.38	<.001	70.4 ± 10.97	62.0 ± 10.00	<.001	.008	.008	<.001	.003
BMI (kg/m^2^)	25.1 ± 4.02	22.34 ± 3.47	<.001	26.0 ± 3.56	24.8 ± 3.66	<.001	24.81 ± 3.60	25.06 ± 3.85	.43	<.001	<.001	<.001	.77
Abdominal circumference (cm)	86.4 ± 11.59	75.4 ± 9.33	<.001	92.3 ± 10.12	84.4 ± 10.07	<.001	90.77 ± 10.09	88.0 ± 10.51	<.001	<.001	<.001	<.001	<.001
Mid‐upper arm circumference (cm)	29.1 ± 3.06	25.7 ± 3.09	<.001	29.4 ± 2.77	27.8 ± 3.31	<.001	28.09 ± 3.45	27.46 ± 3.23	<.001	<.001	<.001	<.001	.004
Calf circumference (cm)	38.1 ± 3.57	34.6 ± 3.22	<.001	37.0 ± 3.33	34.9 ± 3.22	<.001	35.3 ± 3.51	33.95 ± 3.38	<.001	<.001	.007	<.001	<.001
RSMMI (kg/m^2^)	8.1 ± 0.76	6.2 ± 0.66	<.001	7.8 ± 0.79	6.4 ± 0.70	<.001	7.5 ± 0.79	6.2 ± 0.72	<.001	<.001	.078	<.001	.001
Limb muscle mass (kg)	24.8 ± 3.23	16.3 ± 2.08	<.001	23.2 ± 3.23	16.6 ± 2.34	<.001	21.3 ± 3.20	15.5 ± 2.40	<.001	<.001	<.001	<.001	<.001
Fat free mass (kg)	59.0 ± 7.45	40.5 ± 4.41	<.001	55.0 ± 6.82	41.0 ± 4.65	<.001	50.8 ± 6.56	39.3 ± 4.75	<.001	<.001	<.001	<.001	.001
Muscle mass (kg)	55.7 ± 6.98	38.1 ± 4.15	<.001	52.0 ± 6.49	38.6 ± 4.43	<.001	48.0 ± 6.23	37.0 ± 4.53	<.001	<.001	<.001	<.001	.001
Fat mass (kg)	17.8 ± 8.68	18.2 ± 6.53	.049	20.7 ± 7.26	22.5 ± 7.13	.002	19.9 ± 7.21	22.7 ± 7.17	<.001	<.001	<.001	<.001	.79
Fat percentage (%)	22.2 ± 7.88	30.2 ± 6.29	<.001	26.8 ± 6.69	34.6 ± 6.01	<.001	27.4 ± 7.48	35.9 ± 6.93	<.001	<.001	<.001	<.001	.11
Handgrip strength (kg)	48.4 ± 9.00	27.2 ± 4.85	<.001	39.5 ± 9.57	25.2 ± 5.15	<.001	31.0 ± 9.06	20.5 ± 5.75	<.001	<.001	<.001	<.001	<.001
Speed (S)	1.4 ± 0.44	1.3 ± 0.33	.002	1.2 ± 0.34	1.2 ± 0.39	.44	1.03 ± 0.35	1.0 ± 0.40	.014	<.001	<.001	<.001	<.001
Balance (S)	31.5 ± 56.11	20.9 ± 27.16	<.001	7.9 ± 6.92	10.3 ± 10.82	.087	4.5 ± 5.62	4.8 ± 3.91	.076	<.001	<.001	<.001	<.001
EQ‐5D	0.96 ± 0.81	0.94 ± 0.10	<.001	0.95 ± 0.10	0.93 ± 0.11	.008	0.95 ± 0.10	0.95 ± 0.17	.28	.037	.042	.66	.013
MMSE	29.8 ± 1.0	29.9 ± 0.68	.12	28.5 ± 2.77	29.0 ± 1.91	.029	26.9 ± 3.76	27.0 ± 3.62	.78	<.001	<.001	<.001	<.001
ADL	100.0 ± 0.0	100.0 ± 0.0	1.0	99.8 ± 2.96	100.0 ± 0.24	.17	99.4 ± 5.82	99.5 ± 5.97	.16	<.001	.031	<.001	.024
Exercise, n (%)	340 (82.9)	294 (54.6)	<..001	121 (65.1)	315 (73.8)	.029	403 (68.1)	640 (68.4)	.88	.20	.073	.37	.24
IPAQ^f^, n (%)													
High intensity	156 (38.33)	115 (21.4)	<.001	33 (17.7)	86 (20.3)	.56	65 (11.1)	101 (10.9)	.59	<.001	<.001	<.001	<.001
Moderate intensity	179 (44.98)	251 (46.7)		107 (57.5)	248 (48.5)		385 (65.6)	585 (63.4)					
Low intensity	72 (17.69)	172 (32.0)		46 (24.7)	90 (21.2)		137 (23.3)	237 (25.7)					

Abbreviations: ADL, activities of daily; EQ‐5D, 5‐dimensional EuroQo; MMSE, Mini‐Mental State Examination; RSMM, relative skeletal muscle mass index.

^a^ The *P* value indicated the significance between sex in each age group.

^b^ The significance was presented among three age group.

^c^ The *P* value indicated the significance between young and middle age adults, the significance was adjusted by .0167.

^d^ The *P* value indicated the significance between young and elderly adults, the significance was adjusted by .0167.

^e^ The P value indicated the significance between middle age and elderly adults, the significance was adjusted by .0167.

^f^ International Physical Activity Questionnaire (IPAQ) was used to evaluate activity level in all elder subjects.

* Continuous variables *P* values were calculated by the Mann‐Whitney U test.

** Categorical variables *P* values were calculated by the chi‐squared test.

According to the EWGSOP and AWGS (2014) definition and cut‐off points, sarcopenia is present in subjects with reduced muscle mass and low muscle function (strength or performance). Hence, 11.6% of our study population presented with sarcopenia, in which 10.3% subjects were men and 12.4% subjects were women (Table 2). We further observed that 10.1% participants had pre‐sarcopenia, and 4.7% participants had severe sarcopenia. Men suffered pre‐sarcopenia and severe sarcopenia more frequently than women. According to the EWGSOP2 (2019) cut‐off points, only 5.7% of our study population had sarcopenia.

**TABLE 2:** Prevalence of sarcopenia based on different diagnostic criteria

	Pre‐sarcopenia (2014) or probable sarcopenia (2019)				Sarcopenia				Severe sarcopenia				*P*_2_ ^b^ value	All Sarcopenia
	Total	Male	Female	*P*_1_ ^a^ value	Total	Male	Female	*P*_1_ *‐*value	Total	Male	Female	*P*_1_‐value		Total
EWGSOP + AWGS^c^														
Ag e ≥ 60 years	154 (10.1%)	77 (13.0%)	77 (8.2%)	.002	177 (11.6%)	61 (10.3%)	116 (12.4%)	.21	71 (4.7%)	38 (6.4%)	33 (3.5%)	.009	<.001	401 (26.3%)
Age 60‐69 years	89 (9.7%)	43 (12.3%)	46 (8.1%)	.034	78 (8.5%)	23 (6.6%)	55 (9.7%)	.11	10 (1.1%)	8 (2.3%)	2 (0.4%)	.008	<.001	177 (19.3%)
Age 70‐79 years	54 (13.4%)	26 (16.6%)	28 (11.3%)	.13	56 (13.9%)	22 (14.0%)	34 (13.8%)	.94	26 (6.4%)	12 (7.6%)	14 (5.7%)	.43	.001	136 (33.7%)
Age ≥ 80 years	11 (5.4%)	8 (9.3%)	3 (2.5%)	.033	43 (21.0%)	16 (18.6%)	27 (22.7%)	.48	35 (17.1%)	18 (20.9%)	17 (14.3%)	.21	<.001	89 (43.5%)
EWGSOP2‐revised														
Age ≥ 60 years	395 (25.9%)	193 (32.6%)	202 (21.6%)	<.001	87 (5.7%)	47 (7.9%)	40 (4.3%)	.003	54 (3.5%)	40 (6.7%)	14 (1.5%)	<.001	<.001	536 (35.1%)
Age 60‐69 years	173 (18.8%)	79 (22.6%)	94 (16.5%)	.021	33 (3.6%)	16 (4.6%)	17 (3.0%)	.21	10 (1.1%)	10 (2.9%)	0 (0%)	<.001	<.001	216 (23.5%)
Age 70‐79 years	116 (28.7%)	60 (38.2%)	56 (22.7%)	.001	28 (6.9%)	17 (10.8%)	11 (4.5%)	.014	17 (4.2%)	12 (7.6%)	5 (2.0%)	.006	<.001	159 (39.8%)

^a^ The *P*_1_ value indicated the significance between different sex in each age group.

^b^ The *P*_2_ value indicated the significance of the three sarcopenia stages among different age groups.

^c^ 2014 indicates the sarcopenia was diagnosed with the 2014 version of the EWGSOP pathway and the 2014 AWGS cutoffs; EWGSOP2 indicates the sarcopenia was diagnosed with the 2019 version of the EWGSOP2 diagnostic criteria; EWGSOP2‐revised indicates the sarcopenia was diagnosed based on the EWGSOP2 and modified the skeletal muscle mass index for females (5.5 kg/m^2^); EWGSOP, The European Working Group in Sarcopenia in Older Adults; AWGS, the Asian Working Group for Sarcopenia.

We also considered the prevalence of sarcopenic obesity using four different definitions of sarcopenia (Table 3). According to the Baumgartner definition, the prevalence of sarcopenic obesity was 4.1%, and 5.8%, respectively, based on two definitions of obesity: P_60_ of fat percentage and WHO reference fat percentage cut‐off points.^20^ According to the EWGSOP and AWGS (2014) definition, the prevalence of sarcopenic obesity was 6.0%, and 9.1% respectively, based on the two definitions of obesity. According to the EWGSOP2 (2019) definition, the prevalence of sarcopenic obesity was 3.6%, and 5.8%, respectively. The prevalence of sarcopenic obesity, as defined by BMI, approached zero in both male and female, suggesting that BMI might not be appropriate for defining sarcopenic obesity. The most robust definition for sarcopenic obesity appeared to be based on body fat percentage, ranging from 3.6% to 9.1% for various definitions of sarcopenia. The EWGSOP and AWGS (2014) definition gave the highest percentage of participants with sarcopenic obesity.

**TABLE 3:** The prevalence of sarcopenic obesity based on different criteria obesity and sarcopenia definitions

	Abdominal circumference (male ≥ 90 cm, female ≥ 85 cm)				60th percentile of fat percentage (male = 29.6%, female = 37.9%)				Fat percentage (male ≥ 25% female ≥ 35%)				BMI ≥ 28 kg/m^2^			
	Male	Female	Total	*P* value^a^	Male	Female	Total	*P* value	Male	Female	Total	*P* value	Male	Female	Total	*P* value
2014^b^	32 (5.41%)	56 (5.98%)	88 (5.76%)	.64	43 (7.26%)	49 (5.24%)	92 (6.02%)	.10	65 (11.0%)	74 (7.91%)	139 (9.10%)	.042	2 (0.34%)	4 (0.43%)	6 (0.39%)	1.0
2019	27 (4.56%)	20 (2.14%)	47 (3.08%)	.008	36 (6.08%)	19 (2.03%)	55 (3.60%)	<.001	56 (9.46%)	32 (3.42%)	88 (5.76%)	<.001	2 (0.34%)	2 (0.21%)	4 (0.26%)	.64
Baumgartner	27 (4.56%)	11 (1.18%)	38 (2.49%)	<.001	43 (7.26%)	20 (2.14%)	63 (4.12%)	<.001	63 (10.64%)	26 (2.78%)	89 (5.82%)	<.001	7 (1.18%)	1 (0.11%)	8 (0.52%)	.005

^a^ The *P* value indicated the significance between different sex in each age group.

^b^ 2014 indicates the sarcopenia was diagnosed with the 2010 version EWGSOP pathway and 2014 AWGS cutoffs; 2018 indicates the sarcopenia was diagnosed with the 2018 version of the EWGSOP2 diagnostic criteria; 2019 indicates the sarcopenia was diagnosed with the 2019 version of the EWGSOP2 cutoff value for RSSMI; Baumgartner indicates sarcopenia was diagnosed as muscle mass > 2SD below the young adults’ mean value.

### 3.4 Demographic risk factors for sarcopenia

We chose the EWGSOP and AWGS (2014) definition for further analysis of the risk factors for sarcopenia, because its cut‐off points have been optimized with Asians and could most robustly identify sarcopenia in our study population. A comparison of the demographic characteristics of sarcopenic and non‐sarcopenic participants, based on the EWGSOP and AWGS (2014) definition, is shown in Tables 4 and 5. Sarcopenic participants were significantly older than non‐sarcopenic participants on average (74 years vs 68 years, *P* < .001), as expected based on previous observations that sarcopenia progresses with normal ageing (Table 4). After adjustments for other demographic parameters and socioeconomic status, multivariate analysis showed that age alone was a significant predictor of sarcopenic risk and the risk level increased with ageing (OR = 2.301, 95% CI [1.530, 3.461], *P* < .001 for 70‐79 years; OR = 5.253, 95% CI [3.174, 8.695], *P* < .001 for ≥80 years).

**TABLE 4:** Descriptive characteristics of participants by sarcopenia status based on the AWGS (2014) cutoff points among elderly subjects ≥60 years

	Male			Female			Total		
	Sarcopenia, mean ± SD, n (%)	Non‐sarcopenia, mean ± SD/n(%)	*P* value^a^	Sarcopenia, mean ± SD/n (%)	Non‐sarcopenia, mean ± SD/n (%)	*P* value	Sarcopenia, mean ± SD/n (%)	Non‐sarcopenia, mean ± SD/n (%)	*P* value
N	99 (16.7)	493 (83.3)		149 (15.9)	787 (84.1)		248 (16.2)	1280 (83.8)	
Age (years)	75.34 ± 8.71	68.48 ± 7.10	<.001	73.80 ± 9.05	68.18 ± 6.99	<.001	74.42 ± 9.93	68.29 ± 7.03	<.001
Education level, n (%)									
Master or above	1 (1.0)	9 (1.8)	.65	3 (2.0)	14 (1.8)	.99	4 (1.6)	23 (1.8)	.83
Bachelor or Junior college	27 (27.3)	109 (22.1)		21 (14.1)	131 (16.6)		48 (19.4)	240 (18.8)	
High or secondary school	18 (18.2)	96 (19.5)		34 (22.8)	176 (22.4)		52 (21.0)	272 (21.3)	
Junior middle school	34 (34.3)	190 (38.5)		53 (35.6)	247 (31.4)		87 (35.1)	437 (34.1)	
Primary school or lower	19 (19.2)	89 (18.1)		38 (25.5)	219 (27.8)		57 (23.0)	308 (24.1)	
Living situation, n (%)·									
Living with three generations	4 (4.0)	30 (6.1)	.06	10 (6.7)	44 (5.6)	.019	14 (5.6)	74 (5.8)	.001
Living with spouse and kids	18 (18.2)	115 (23.4)		23 (15.4)	189 (24.0)		41 (16.5)	304 (23.8)	
Living with parents	3 (3.0)	13 (2.6)		7 (4.7)	46 (5.9)		10 (4.0)	59 (4.6)	
Living with spouse	48 (48.5)	263 (53.5)		69 (46.3)	376 (47.8)		117 (47.2)	639 (50.0)	
Alone	26 (26.3)	71 (14.4)		40 (26.8)	131 (16.7)		66 (26.6)	202 (15.8)	
Working type, n (%)									
Relative high intensity	2 (2.0)	10 (2.0)	1.0	0 (0)	11 (1.4)	.23	2 (0.8)	21 (1.6)	.57
Relative low intensity	97 (98.0)	483 (98.0)		149 (100)	776 (98.6)		246 (99.2)	1259 (98.4)	
Income, n (%)									
More than $732.12	22 (22.2)	63 (12.8)	.047	18 (12.2)	57 (7.3)	.069	40 (16.2)	120 (9.4)	.008
$585.69‐$732.12	18 (18.2)	80 (16.2)		16 (10.8)	119 (15.2)		34 (13.8)	199 (15.6)	
$439.27‐$585.69	18 (18.2)	108 (21.9)		37 (25.0)	155 (19.8)		55 (22.3)	263 (20.6)	
$292.85‐$439.27	24 (24.2)	144 (28.2)		51 (34.5)	249 (31.8)		75 (30.4)	393 (30.8)	
Less than $292.85	17 (17.2)	98 (19.9)		26 (17.6)	204 (26.0)		43 (17.4)	302 (23.6)	
Marital status, n (%)									
Married	71 (71.7)	424 (86.2)	.001	83 (55.7)	590 (75.0)	<.001	154 (62.1)	1014 (79.3)	<.001
Separated	2 (2.0)	11 (2.2)		6 (4.0)	22 (2.8)		8 (3.2)	33 (2.6)	
Divorced	1 (1.0)	6 (1.2)		2 (1.3)	20 (2.5)		3 (1.2)	26 (2.0)	
Widowed	25 (25.3)	51 (10.4)		58 (38.9)	155 (19.7)		83 (33.5)	206 (16.1)	
Smoking, n (%)									
Never	56 (56.6)	273 (55.4)	.84	138 (92.6)	762 (96.8)	.013	194 (78.2)	1035 (80.9)	.31
Former	19 (19.2)	98 (19.9)		2 (1.3)	9 (1.1)		21 (8.5)	107 (8.4)	
Current	24 (24.2)	122 (24.7)		9 (6.0)	16 (2.0)		23 (13.3)	138 (10.8)	
Alcohol drinking, n (%)									
Never	70 (70.7)	306 (62.1)	.16	138 (92.6)	737 (93.6)	.67	208 (83.9)	1043 (81.5)	.37
Once or twice a month	3 (3.0)	35 (7.1)		5 (3.4)	21 (2.7)		8 (3.2)	56 (4.4)	
Once or twice a week	11 (11.1)	66 (13.4)		6 (4.0)	18 (2.3)		17 (6.9)	84 (6.6)	
Almost every day	15 (15.2)	86 (17.4)		0 (0)	11 (1.4)		15 (6.0)	97 (7.6)	
Nutrition status, n (%)									
Malnutrition risk	42 (43.3)	184 (37.3)	.001	85 (57.4)	328 (41.7)	<.001	127 (51.8)	512 (40.0)	<.001
Malnutrition	12 (12.4)	14 (2.8)		9 (6.1)	13 (1.7)		21 (8.6)	27 (2.1)	
IPAQ, n (%)									
High intensity	5 (5.1)	60 (12.3)	.039	5 (3.4)	96 (12.4)	.22	10 (4.1)	156 (12.3)	.025
Moderate intensity	65 (66.3)	320 (65.4)		106 (72.1)	479 (61.7)		171 (69.8)	799 (63.2)	
Low intensity	28 (28.6)	109 (22.3)		36 (24.5)	201 (25.9)		64 (26.1)	310 (24.5)	
Comorbidities, n (%)									
Diabetes	19 (19.2)	79 (16.0)	.44	26 (17.4)	150 (19.1)	.65	45 (18.1)	229 (17.9)	.92
Coronary heart disease	26 (34.2)	56 (18.7)	.004	29 (22.0)	130 (22.2)	.96	55 (26.4)	186 (21.0)	.089
Hypertension	40 (52.6)	133 (44.5)	.20	48 (36.4)	280 (47.8)	.017	88 (42.3)	413 (46.7)	.26
Hyperlipidemia	16 (21.1)	65 (21.7)	.90	30 (22.7)	214 (36.6)	.002	46 (22.1)	279 (31.6)	.007
Osteoarthritis	7 (9.2)	33 (11.0)	.65	26 (19.7)	150 (25.6)	.15	33 (15.9)	183 (20.7)	.12
Osteoporosis	17 (17.2)	49 (9.9)	.037	47 (31.5)	170 (21.6)	.008	64 (25.8)	219 (17.1)	.001
Fractures	8 (8.1)	28 (5.7)	.36	32 (21.5)	89 (11.3)	.001	40 (16.1)	117 (9.1)	.001
Respiratory disease	5 (6.6)	22 (7.4)	.82	9 (6.8)	38 (6.5)	.89	14 (6.7)	60 (6.8)	.98
Cancer	2 (2.0)	24 (4.9)	.29	10 (6.7)	22 (2.8)	.016	12 (4.8)	46 (3.6)	.35
Digestive disease	6 (7.9)	29 (9.7)	.63	19 (14.4)	65 (11.1)	.29	25 (12.0)	94 (10.6)	.56
Renal dysfunction	5 (6.6)	19 (6.4)	1.0	7 (5.3)	33 (5.6)	.88	12 (5.8)	52 (5.9)	.95
Hepatic dysfunction	2 (2.6)	9 (3.0)	1.0	2 (1.5)	31 (5.3)	.061	4 (1.9)	40 (4.5)	.086
BMI (kg/m^2^)	22.28 ± 3.15	25.32 ± 3.47	<.001	22.10 ± 3.05	25.62 ± 3.73	<.001	22.17 ± 3.08	25.50 ± 3.63	<.001
Abdominal circumference (cm)	84.72 ± 8.72	92.17 ± 9.90	<.001	81.83 ± 9.54	89.17 ± 10.28	<.001	82.98 ± 9.31	90.32 ± 10.23	<.001
Mid‐upper arm circumference (cm)	25.65 ± 3.48	28.58 ± 3.23	<.001	25.27 ± 2.93	27.87 ± 3.12	<.001	25.42 ± 3.16	28.15 ± 3.18	<.001
Calf circumference (cm)	32.56 ± 2.66	35.84 ± 3.41	<.001	31.07 ± 2.95	34.50 ± 3.17	<.001	31.66 ± 2.92	35.02 ± 3.33	<.001
RSMMI (kg/m^2^)	6.46 ± 0.46	7.67 ± 0.68	<.001	5.27 ± 0.37	6.41 ± 0.62	<.001	5.75 ± 0.71	6.89 ± 0.89	<.001
Fat free mass (kg)	43.37 ± 4.02	52.24 ± 5.94	<.001	33.81 ± 3.09	40.31 ± 4.27	<.001	37.62 ± 5.84	44.91 ± 7.65	<.001
Muscle mass (kg)	40.76 ± 3.95	49.41 ± 5.57	<.001	31.68 ± 3.00	37.98 ± 4.05	<.001	35.31 ± 5.61	42.38 ± 7.28	<.001
Fat mass (kg)	17.56 ± 6.23	20.41 ± 7.31	<.001	18.47 ± 5.83	23.51 ± 7.12	<.001	18.11 ± 6.01	23.32 ± 7.34	<.001
Fat percentage (%)	28.03 ± 8.06	27.25 ± 7.36	.29	34.67 ± 7.68	36.15 ± 6.75	.03	32.02 ± 8.47	32.72 ± 8.23	.24
Handgrip strength (kg)	21.94 ± 5.57	32.77 ± 8.54	<.001	15.96 ± 3.51	21.38 ± 5.68	<.001	18.35 ± 5.32	25.76 ± 8.87	<.001
Balance (s)	3.09 ± 4.03	4.77 ± 5.82	.03	2.71 ± 2.68	5.21 ± 15.03	.001	2.88 ± 3.32	5.01 ± 11.90	<.001
Appetite									
Strong	29 (29.6)	160 (32.5)	.51	29 (19.5)	224 (28.5)	.009	58 (23.5)	384 (30)	.016
General	66 (67.3)	323 (65.5)		114 (76.5)	551 (70.0)		180 (72.9)	874 (68.3)	
Poor	3 (3.1)	10 (2.0)		6 (4.0)	12 (1.5)		9 (3.6)	22 (1.7)	
Protein distribution									
Three meals	42 (42.9)	202 (41.1)	.52	80 (54.1)	347 (44.1)	.069	122 (49.6)	549 (43.0)	.07
Two meals	46 (46.9)	220 (44.8)		45 (30.4)	312 (39.7)		91 (37.0)	532 (41.7)	
One meal	10 (10.2)	69 (14.1)		23 (15.5)	127 (16.2)		33 (13.4)	196 (15.3)	
Protein intake (g/day)									
Total protein	45.67 ± 16.65	46.22 ± 17.95	.93	41.06 ± 13.93	44.61 ± 14.27	.005	42.90 ± 15.21	45.23 ± 15.81	.033
Animal protein	21.12 ± 12.10	21.51 ± 11.85	.73	19.11 ± 9.22	21.41 ± 10.48	.018	19.92 ± 10.49	21.45 ± 11.03	.044
Vegetable protein	24.36 ± 10.01	24.81 ± 10.67	.88	22.10 ± 8.41	23.30 ± 8.45	.10	23.01 ± 9.13	23.88 ± 9.39	.19
Fish frequency^b^									
Almost everyday	3 (3.1)	21 (4.3)	.52	5 (3.4)	36 (4.6)	.59	8 (3.3)	57 (4.5)	1.0
Less once every week	78 (79.6)	363 (74.1)		117 (79.1)	620 (78.9)		195 (79.3)	983 (77.0)	
None	17 (17.3)	106 (21.6)		26 (17.6)	130 (16.5)		43 (17.5)	236 (18.5)	
Poultry frequency									
Almost everyday	10 (10.2)	52 (10.6)	.63	10 (6.8)	66 (8.4)	.69	20 (8.1)	118 (9.2)	.55
Less once every week	71 (72.4)	364 (74.3)		113 (76.4)	589 (74.9)		184 (74.8)	953 (74.7)	
None	17 (17.3)	74 (15.1)		25 (16.9)	131 (16.7)		42 (17.1)	205 (16.1)	
Meat frequency^c^									
Almost everyday	41 (41.8)	256 (52.2)	.03	43 (29.1)	351 (44.7)	.001	84 (34.1)	607 (47.6)	<.001
Less once every week	46 (46.9)	205 (41.8)		91 (61.5)	374 (47.6)		137 (55.7)	579 (45.4)	
None	11 (11.2)	29 (5.9)		14 (9.5)	61 (7.8)		25 (10.2)	90 (7.1)	
Milk frequency^d^									
Almost everyday	46 (46.9)	183 (37.3)	.089	69 (46.6)	382 (48.6)	.91	115 (46.7)	565 (44.3)	.33
Almost every week	19 (19.4)	107 (21.8)		36 (24.3)	163 (20.7)		55 (22.4)	270 (21.2)	
None	33 (33.7)	200 (40.8)		43 (29.1)	241 (30.7)		76 (30.9)	441 (34.6)	
Diary product									
Milk	56 (57.1)	239 (48.8)	.22	87 (58.8)	406 (51.7)	.37	143 (58.1)	645 (50.5)	.29
Yogurt	7 (7.1)	42 (8.6)		19 (12.8)	122 (15.5)		26 (10.6)	164 (12.9)	
Cheese	5 (5.1)	15 (3.1)		2 (1.4)	31 (3.9)		7 (2.8)	46 (3.6)	
Milk powder	0 (0)	6 (1.2)		2 (1.4)	11 (1.4)		2 (0.8)	17 (1.3)	
None	30 (30.6)	188 (38.4)		38 (25.7)	216 (27.5)		68 (27.6)	404 (31.7)	
Bean frequency									
Almost everyday	28 (28.6)	200 (40.8)	.001	36 (24.3)	258 (32.8)	.012	64 (26.0)	458 (35.9)	<.001
Less once every week	50 (51.0)	250 (51.0)		87 (58.8)	441 (56.1)		137 (55.7)	691 (54.2)	
None	20 (20.4)	40 (8.2)		25 (16.9)	87 (11.1)		45 (18.3)	127 (10.0)	
Nut frequency									
Almost everyday	18 (18.4)	116 (23.6)	.092	36 (24.3)	288 (36.6)	.032	54 (22.0)	404 (31.6)	.008
Less once every week	25 (25.5)	146 (29.7)		57 (38.5)	234 (29.8)		82 (33.3)	380 (29.7)	
None	55 (56.1)	230 (26.7)		55 (37.2)	264 (33.6)		110 (44.7)	494 (38.7)	
Grain intake (g/day)·									
≥300g	16 (16.3)	117 (23.9)	.16	13 (8.8)	119 (15.2)	.34	29 (11.8)	236 (18.5)	.11
≥200g and < 300g	38 (38.8)	183 (37.3)		59 (39.9)	279 (35.5)		97 (39.4)	462 (36.2)	
≥100g and < 200g	34 (34.7)	138 (28.2)		60 (40.5)	301 (38.3)		94 (38.2)	439 (34.4)	
<100g	10 (10.2)	52 (10.6)		16 (10.8)	86 (11.0)		26 (10.6)	138 (10.8)	
Milk intake (mL/meal)									
≥500 mL	0 (0)	11 (2.2)	.051	4 (2.7)	21 (2.7)	.69	4 (1.6)	32 (2.5)	.36
≥250 and <500 mL	35 (35.7)	116 (23.7)		40 (27.0)	245 (31.2)		75 (30.5)	361 (28.3)	
<250 mL	34 (34.7)	175 (35.7)		65 (43.9)	302 (38.4)		99 (40.2)	477 (37.4)	
0 mL	29 (29.6)	188 (38.4)		39 (26.4)	218 (27.7)		68 (27.6)	406 (31.8)	
Fish intake (g/meal)									
≥150 g	13 (13.3)	67 (13.7)	.9	14 (9.5)	102 (13.0)	.024	27 (11.0)	169 (13.2)	.071
≥100 g and < 150 g	22 (22.4)	100 (20.4)		27 (18.2)	199 (25.3)		49 (19.9)	299 (23.4)	
≥50 g and < 100 g	29 (29.6)	164 (33.5)		58 (39.2)	270 (34.4)		87 (35.4)	434 (34.0)	
No	34 (34.7)	159 (32.4)		49 (33.1)	215 (27.4)		83 (33.7)	374 (29.3)	
Meat intake (g/day)·									
≥150 g	2 (2.1)	30 (6.1)	.011	7 (4.7)	37 (4.7)	.015	9 (3.7)	67 (5.3)	.001
≥100 g and < 150 g	20 (20.6)	152 (31.1)		26 (17.6)	207 (26.3)		46 (18.8)	359 (28.2)	
≥50 g and < 100 g	39 (40.2)	162 (33.1)		51 (34.5)	277 (35.2)		90 (36.7)	439 (34.4)	
0 g	36 (37.1)	145 (29.7)		64 (43.2)	265 (33.7)		100 (40.8)	410 (32.2)	
Poultry intake (g/meal)									
≥150 g	5 (5.1)	38 (7.8)	.30	7 (4.7)	43 (5.5)	.038	12 (4.9)	81 (6.3)	.024
≥100 g and < 150 g	23 (23.5)	112 (22.9)		24 (16.2)	199 (25.3))		47 (19.1)	311 (24.4)	
≥50 g and < 100 g	38 (38.8)	209 (42.7)		66 (44.6)	313 (39.8)		104 (42.3)	522 (40.9)	
0 g	32 (32.7)	131 (26.7)		51 (34.5)	231 (29.4)		83 (33.7)	362 (28.4)	
Vegetable intake (g/day)									
≥500 g	17 (17.3)	142 (28.9)	.14	26 (17.6)	243 (31.0)	.003	43 (17.5)	385 (30.3)	.001
≥250 and < 500 g	53 (54.1)	210 (42.8)		70 (47.3)	321 (40.9)		123 (50.0)	531 (41.6)	
<250 g	24 (24.5)	125 (25.5)		49 (33.1)	207 (26.4)		73 (29.7)	332 (26.0)	
0 g	4 (4.1)	14 (2.9)		3 (2.0)	14 (1.8)		7 (2.8)	28 (2.2)	
Fruit intake (g/day)									
≥500 g	10 (10.2)	48 (9.8)	.2	18 (12.2)	102 (13.0)	.93	28 (11.4)	150 (11.8)	.46
≥250 and < 500 g	33 (33.7)	202 (41.1)		63 (42.6)	318 (40.5)		96 (39.0)	520 (40.8)	
<250 g	36 (36.7)	171 (34.8)		49 (33.1)	270 (34.4)		85 (34.6)	441 (34.6)	
0 g	19 (19.4)	70 (14.3)		18 (12.2)	95 (12.1)		37 (15.0)	165 (12.9)	
Bean intake (g/meal)									
≥150g	3 (3.1)	40 (8.2)	.03	8 (5.4)	46 (5.9)	.046	11 (4.5)	86 (6.7)	.004
≥100 and < 150 g	38 (38.8)	173 (35.3)		39 (26.4)	262 (33.4)		77 (31.3)	435 (34.1)	
≥50 and < 100 g	20 (20.4)	177 (36.1)		59 (39.9)	308 (39.2)		79 (32.1)	485 (38.0)	
0 g	37 (37.8)	100 (20.4)		42 (28.4)	169 (21.5)		79 (32.1)	269 (21.1)	
Nut intake (g/meal)									
≥50 g	8 (8.2)	61 (12.4)	.061	15 (10.1)	82 (10.4)	.11	23 (9.3)	143 (11.2)	.015
≥20 and < 50 g	17 (17.3)	115 (23.4)		32 (21.6)	254 (32.3)		49 (19.9)	369 (28.9)	
<20 g	18 (18.4)	82 (16.7)		47 (31.8)	185 (23.5)		65 (26.4)	267 (20.9)	
0 g	55 (56.1)	234 (47.6)		54 (36.5)	265 (33.7)		109 (44.3)	499 (39.0)	
Salt intake (g)									
<3	11 (11.2)	42 (8.5)	.53	26 (17.6)	96 (12.2)	.77	37 (15.0)	138 (10.8)	.57
3‐5	44 (44.9)	202 (41.1)		60 (40.5)	357 (45.4)		104 (42.3)	559 (43.7)	
5‐8	24 (24.5)	178 (36.2)		39 (26.4)	249 (31.7)		63 (25.6)	427 (33.4)	
>8	19 (19.4)	70 (14.2)		23 (15.5)	84 (10.7)		42 (17.1)	154 (12.1)	
Oil intake (mL)									
<25	24 (24.5)	102 (20.7)	.81	39 (26.4)	184 (13.4)	.44	63 (25.6)	286 (22.4)	.46
≥25 and <30	56 (57.1)	311 (63.2)		89 (60.1)	486 (61.8)		145 (58.9)	797 (62.4)	
≥30	18 (18.4)	79 (16.1)		20 (13.5)	116 (14.8)		38 (15.4)	195 (15.3)	
Caffeine drinking									
No	54 (55.1)	277 (56.3)	.83	106 (71.6)	532 (67.7)	.35	160 (65.0)	809 (63.3)	.6
Yes	44 (44.9)	215 (43.7)		42 (28.4)	254 (32.3)		86 (35.0)	469 (36.7)	
Supplements, n (%)									
Multivitamin and minerals	8 (8.1)	30 (6.1)	.88	16 (10.7)	73 (9.3)	.81	24 (9.7)	103 (8.0)	.78
Calcium and vitamin D	6 (6.1)	36 (7.3)		18 (12.1)	101 (12.8)		24 (9.7)	137 (10.7)	
Whey protein	2 (2.0)	7 (1.4)		4 (2.7)	14 (1.8)		6 (2.4)	21 (1.6)	
Fish oil	1 (1.0)	9 (1.8)		4 (2.7)	13 (1.7)		5 (2.0)	22 (1.7)	
EQ‐5D	0.94 ± 0.11	0.95 ± 0.1	.12	0.94 ± 0.12	0.95 ± 0.18	.77	0.94 ± 0.12	0.95 ± 0.15	.25
MMSE	26.53 ± 3.86	26.96 ± 3.73	.24	26.47 ± 4.10	27.09 ± 3.52	.058	26.49 ± 4.00	27.04 ± 3.60	.025
ADL	99.59 ± 1.99	99.34 ± 6.31	.056	99.06 ± 6.56	99.54 ± 5.85	.003	99.27 ± 5.25	99.46 ± 6.03	.001

Abbreviations: ADL = activities of daily. EQ‐5D = 5‐dimensional European quality; BMI = body mass index; IPAQ = International Physical Activity Questionnaire; MMSE = Mini‐Mental State Examination; RSMMI = relative skeleton muscle mass index.

^a^ Continuous variables *P* value were calculated by Mann‐Whitney U test; categorical variables *P* values were calculated by the chi‐squared test.

^b^ Almost every day means the frequency more than four times; less once every day means the frequency less than twice every month.

^c^ Almost every day means the frequency more than four times; less once every day means the frequency less than twice every month.

^d^ Almost every day means everyday intake milk; Almost every week means intakes more than three times.

**TABLE 5:** Multivariate analysis of factors associated with sarcopenia

	N	Multivariable model^a^		
		Multivariable odds ratio	95% CI	*P* value
Age (years)				
60‐69	1528	Reference		
70‐79		2.301	1.530, 3.461	<.001
≥80		5.253	3.174, 8.695	<.001
Gender	1528	0.589	0.400, 0.868	.008
Income				
More than $732.12	1528	Reference		
$585.69‐$732.12		0.626	0.332, 1.183	.15
$439.27‐$292.85		1.081	0.624, 1.874	.78
<$292.85		0.904	0.471, 1.736	.76
IPAQ				
High intensity	1528	Reference		
Moderate intensity		2.328	1.108, 4.890	.026
Low intensity		1.713	0.770, 3.813	.19
Marriage				
Married	1528	Reference		
Separated		0.829	0.291, 2.360	.73
Divorced		1.052	0.263, 4.211	.94
Widowed		1.678	1.094, 2.574	.018
BMI (kg/m^2^)				
≥18.5	1528	Reference		
18.5‐24		0.423	0.204, 0.877	.021
24‐28		0.121	0.047, 0.307	<.001
≤28		0.015	0.003, 0.066	<.001
Fat mass (kg)	1528	1.064	1.017, 1.113	.007
Calf circumference (cm)	1528	0.780	0.733, 0.830	<.001
MNA				
Nutrition good	1528	Reference		
Malnutrition risk		1.299	0.886, 1.905	.18
Malnutrition		1.122	0.459, 2.744	.80
Appetite				
Strong	1528	Reference		
General		1.054	0.705, 1.576	.8
Poor		1.376	0.466, 4.065	.56
Total protein (g/d)	1528	1.001	0.987, 1.014	.93
Meat frequency				
Almost everyday	1528	Reference		
Less once one week		1.245	0.854, 1.815	.26
None		1.585	0.790, 3.177	.19
Bean frequency				
Almost everyday	1528	Reference		
Less once 1 week		1.214	0.813, 1.813	.34
None		1.278	0.668, 2.443	.46
Nut frequency				
Almost everyday	1528	Reference		
Less once one week		1.660	1.047, 2.631	.031
None		2.758	0.256, 29.774	.4
Meat intake (g/day)				
≥150 g	1528	Reference		
≥100 and < 150 g		0.861	0.269, 2.757	.8
≥50 and < 100 g		1.332	0.428, 4.139	.62
0 g		1.264	0.400, 3.996	.69
Poultry intake (g/day)				
≥150 g	1528	Reference		
≥100 and < 150 g		1.260	0.441, 3.600	.67
≥50 and < 100 g		1.137	0.412, 3.138	.8
0 g		1.188	0.421, 3.355	.75
Vegetable intake (g/day)				
≥500 g	1528	Reference		
≥250 and < 500 g		1.565	0.987, 2.480	.057
<250 g		1.205	0.711, 2.042	.49
0 g		1.513	0.487, 4.702	.47
Bean intake (g/day)				
≥150 g	1528	Reference		
≥100 and < 150 g		0.924	0.381, 2.245	.86
≥50 and < 100 g		0.639	0.263, 1.550	.32
0 g		1.101	0.432, 2.805	.84
Nut intake (g/day)				
≥50 g	1528	Reference		
≥20 and< 50g		0.561	0.290, 1.083	.085
<20g		0.980	0.516, 1.859	.95
0g		0.406	0.037, 4.486	.46

Abbreviations: BMI, body mass index; IPAQ, International Physical Activity Questionnaire; MNA, mini nutrition assessment.

^a^ The model was included demographics, socioeconomic status, lifestyle, diet intake, anthropometry, and nutritional statues; the *P* value was obtained from the likelihood ratio.

Sarcopenic participants tended towards higher incomes (*P* = .008), living alone without families (*P* = .001), being single (*P* < .001) and suffering from malnutrition risk (*P* < .001), although these associations (Table 4) became less significant after adjustment for other parameters in the multivariate analysis (Table 5).

Although smoking was not associated with sarcopenia in general (Tables 4 and 5), it was more frequent in sarcopenic women (*P* = .013). Similarly, while coronary heart disease and hypertension were not associated with sarcopenia in general (Table 4), they were more frequent in sarcopenic men (*P* = .004) and sarcopenic women (*P* = .017), respectively. Hyperlipidaemia was associated with non‐sarcopenia in general (*P* = .007), especially in women (*P* = .002). Osteoporosis and fracture risks were also associated with sarcopenia in general (*P* = .001), especially in women. Cancer was more frequent in sarcopenic women (*P* = .016). Exercise intensity (IPAQ) and daily activity level (ADL) also showed similarly curious gender‐specific associations.

Interestingly, when adjusted for other parameters in multivariate analysis (Table 5), female participants were greater than twofold less susceptible to sarcopenia than men (OR = 0.589, 95% CI [0.400, 0.868], *P* = .008). This is well reflected in the prevalence of pre‐sarcopenia and severe sarcopenia (Table 2). These results suggest complex interactions between sarcopenia and gender.

### 3.5 Gender‐associated serum risk factors for sarcopenia

**FIGURE 2:**
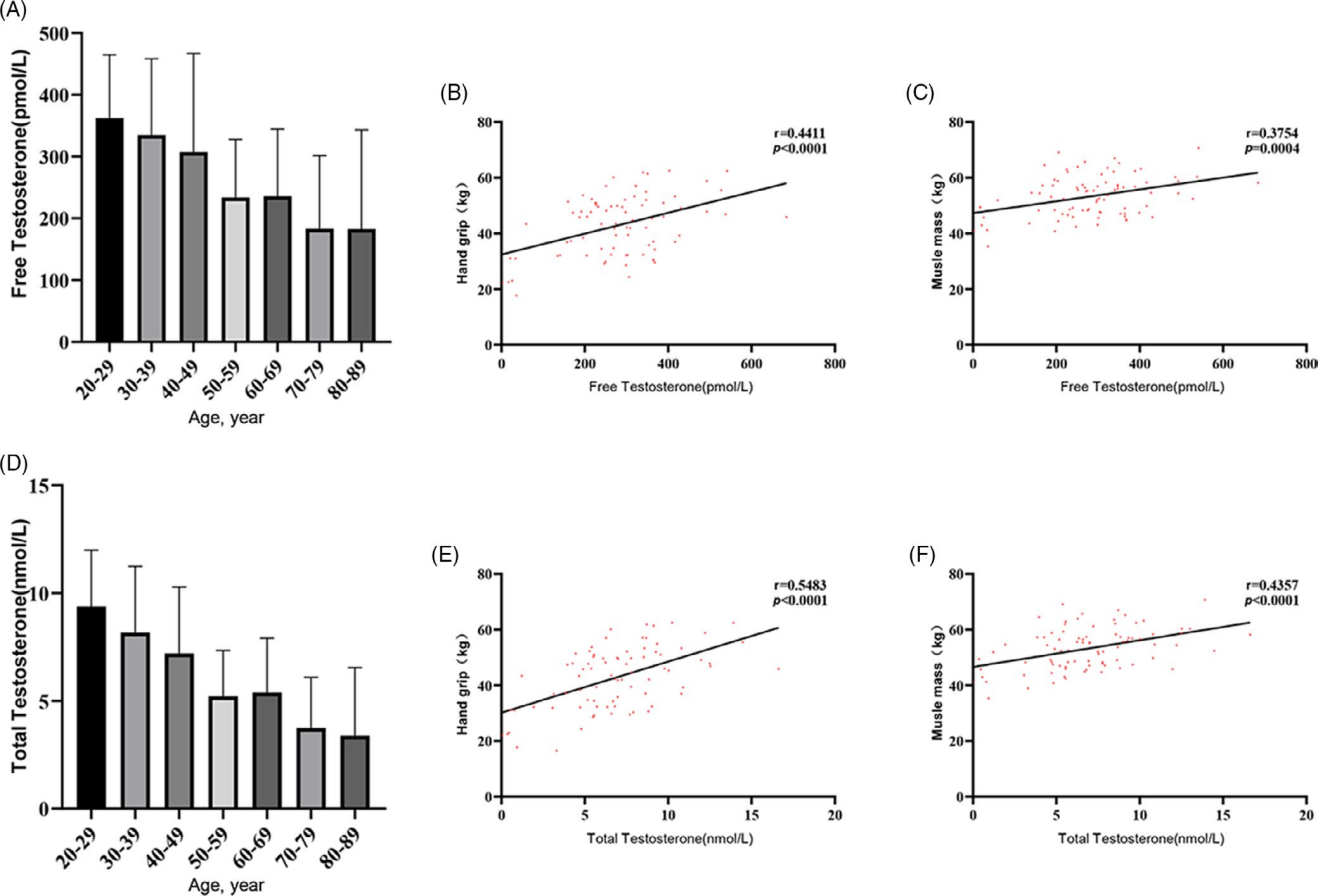
Testosterone levels and muscle parameters in men. Histogram of the testosterone levels in different age groups of male participants. Correlation *r* is adjusted for age and BMI

Complex gender‐specific associations with sarcopenia behoved us to examine the sex‐related sterol hormones more deeply. While elderly women tend to have very low oestradiol levels in general due to menopause, elderly men experience a more gradual drop in testosterone levels at a rate of ~8.2% every 10 years after the age of 30, similar to the rate of muscle decline. Indeed, our study population also reflected a steady decrease in free testosterone with age in men (Figure 2A). Free testosterone levels were significantly correlated with grip strength (*r* = .441, *P* < .001) and muscle mass (*r* = .375, *P* = .004) (Figure 2B,C). These correlations were even stronger if we considered total testosterone, instead of free testosterone (Figure 2D‐F), even though the free (bioactive) testosterone makes up only ~2% to 3% of total testosterone, while the inactive remainder is bound to SHBG or albumin. This suggests that serum testosterone deficiency is more likely to be an effect than a cause for sarcopenia.

**FIGURE 3:**
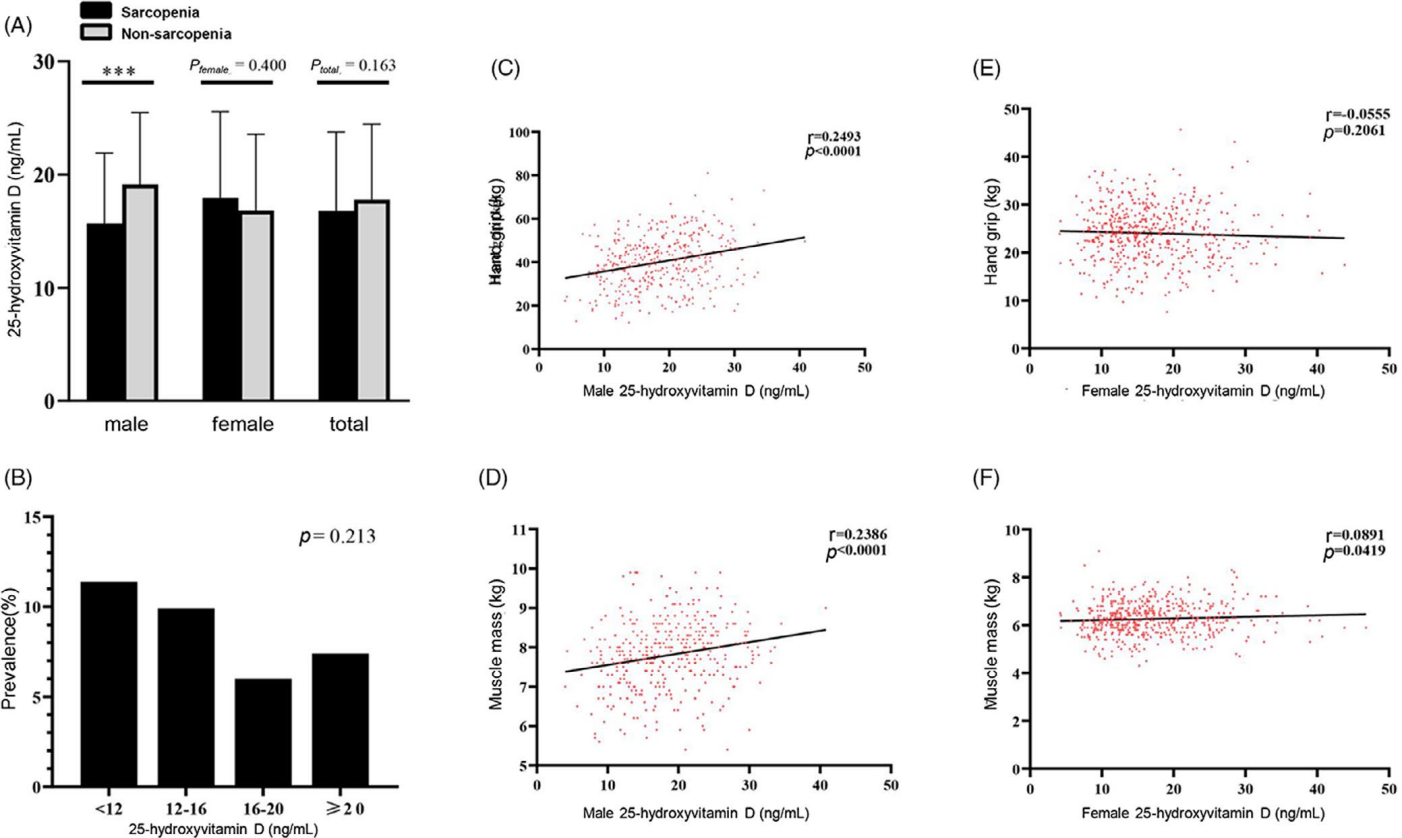
Correlation between serum 25‐hydroxyvitamin D and muscle parameters. Correlation *r* is adjusted for age, gender and BMI. A, Serum levels of 25‐hydroxyvitamin D in sarcopenic and non‐sarcopenic subjects. B, Prevalence of sarcopenia based on the levels of serum 25‐hydroxyvitamin D and the dietary reference intake for vitamin D. C, Correlation between serum 25‐hydroxyvitamin D and hand grip strength in men. D, Correlation between serum 25‐hydroxyvitamin D and muscle mass in men. E, Correlation between serum 25‐hydroxyvitamin D and hand grip strength in women. F, Correlation between serum 25‐hydroxyvitamin D and muscle mass in women

Given that another sterol derivative, cholecalciferol or vitamin D, is known to influence SHBG and testosterone levels, we also examined the serum levels of 25‐hydroxycholecalciferol or 25‐hydroxyvitamin D (25(OH)D). While there were no significant differences in 25(OH)D between sarcopenic and non‐sarcopenic participants in general (17.8 ± 6.68 vs 16.8 ± 6.98 ng/mL, *P* =.163), 25(OH)D was significantly lower in sarcopenic males than non‐sarcopenic males (*P* < .001; Figure 3A). However, female had a greater proportion of 25(OH)D deficiency and serum insufficiency (25.3% and 26.6%) compared with men (15.2% and 20.6%). The prevalence of sarcopenia also tended to decrease as 25(OH)D increased, after adjustment for gender (Figure 3B). In fact, we found that 25(OH)D > 20 ng/mL was associated with fourfold lower odds of sarcopenia in men (OR = 0.224, 95% CI [0.092, 0.544], *P* = .001). There was a significant correlation between 25(OH)D vs grip strength (*r* = .249, *P* < .001) and muscle mass (*r* = .239, *P* < .001) for men (Figure 3C,D), but not for women (Figure 3E,F). These results suggest that men need vitamin D supplementation more than women to protect against sarcopenia, despite the higher rates of 25(OH)D deficiency that we uncovered in Asian Chinese women.

### 3.6 Nutritional and dietary risk factors for sarcopenia

**FIGURE 4:**
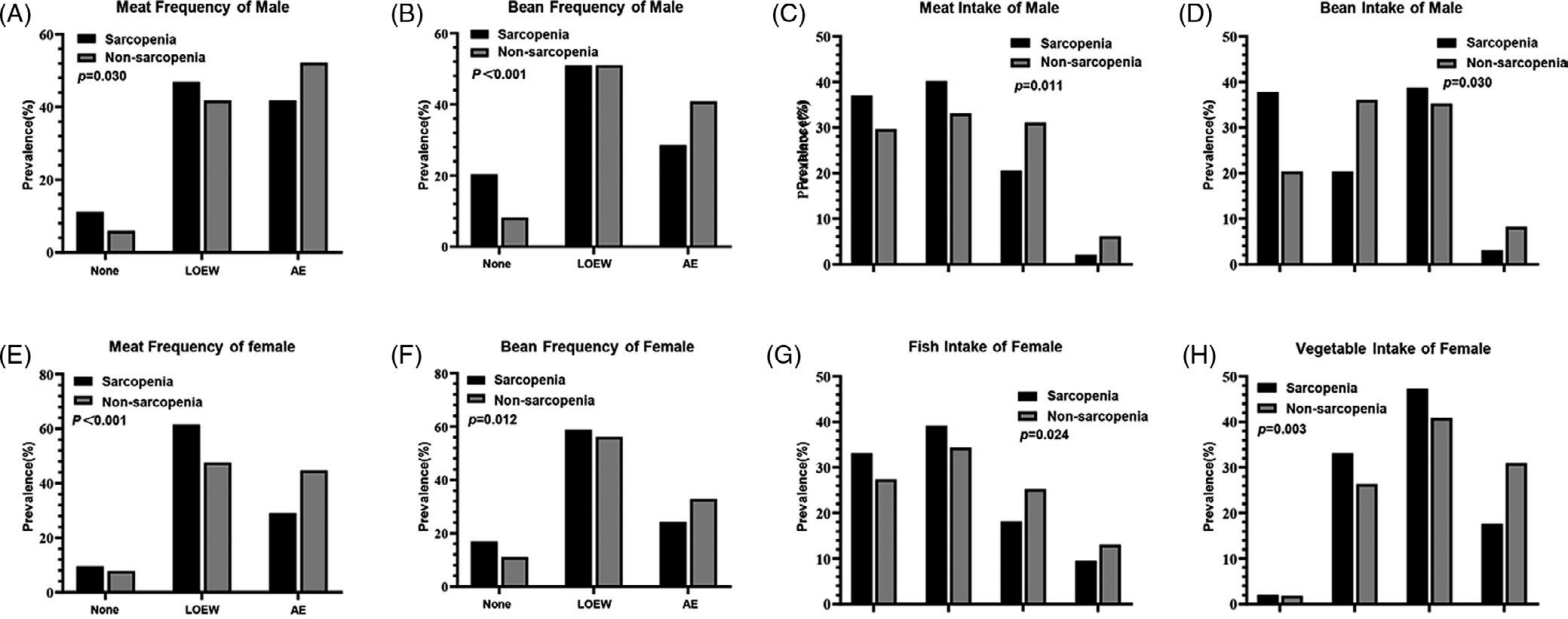
Distribution of important dietary parameters for elderly men and women. Histograms of the dietary parameters statistically significant for men and women

To broadly understand the role of nutrition in sarcopenia, we surveyed the participants’ appetite and intake of various food groups, oil, salt, caffeine and vitamins (Table 4). In general, sarcopenic participants had poor appetite (*P* = .016), lower total and animal protein (*P* = .033 and .044, respectively), lower nut frequency (*P* = .008), lower poultry (only women), vegetable (only women) and nut intake (*P* = .024, *P* = .001 and *P* = .015, respectively) than non‐sarcopenic participants (Table 4). There were again many gender‐specific associations, but both sarcopenic men and women ate less meat and beans (Figure 4). Sarcopenic women tended to have poor appetite (*P* = .009), lower total protein intake (*P* = .005), animal protein intake (*P* = .018), fish intake (*P* = .024), poultry intake (*P* = .038), vegetable intake (*P* = .003) and nut frequency (*P* = .032). Bean intake less than once per week increased the risk for sarcopenia (OR = 1.419, 95% CI [1.031, 1.953], *P* = .032), and this risk further increased with zero bean intake (OR = 2.536, 95% CI [1.651, 3.894], *P* < .001). Similarly, meat intake less than once per week almost doubled the risk for sarcopenia (OR = 1.710, 95% CI [1.274, 2.295], *P* < .001), and this risk further increased with zero meat intake (OR = 2.007, 95% CI [1.219, 3.304], *P* = .006). After adjustment for other parameters in multivariate analysis, it appears that higher nut intake may be protective against sarcopenia (OR = 1.660, 95% CI [1.047, 2.631], *P* = .031).

### 3.7 Associations between body fat and sarcopenia

**FIGURE 5:**
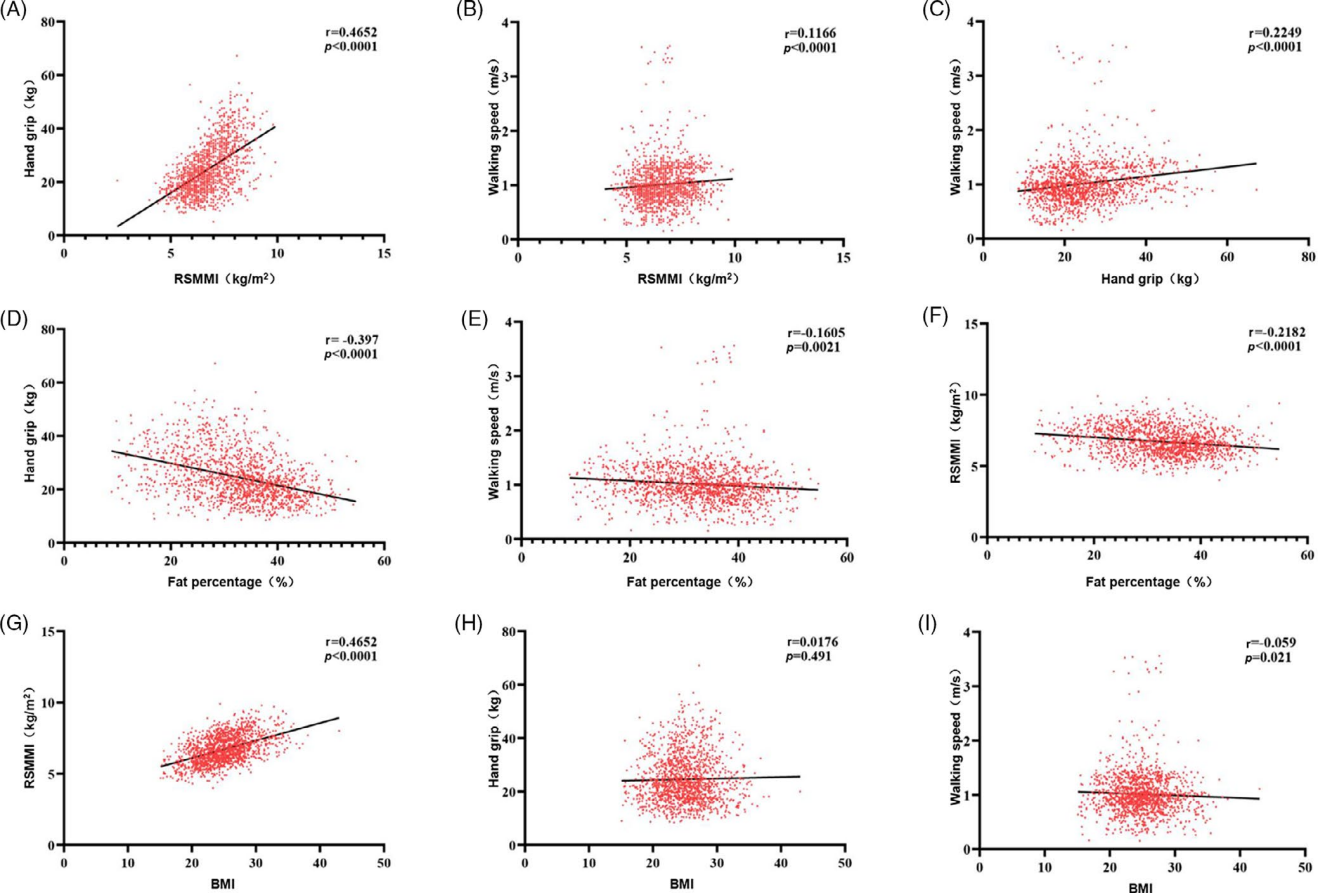
Correlations between body composition parameters and muscle parameters. Correlation *r* is adjusted for age and gender. BMI, body mass index; RSMMI, relative skeletal muscle mass index

Next, we aimed to capture the associations between lean mass, fat mass and other related body composition parameters with sarcopenia (Table 4). As expected, there were significant correlations (Figure 5A,B) between RSMMI vs hand grip strength (*r* = 0.465, *P* < .001), and walking speed (*r* = 0.117, *P* < .001). There was also a significant correlation between hand grip strength and walking speed (*r* = .225, *P* < .001; Figure 5C). In contrast, there were significant inverse correlations between fat percentage and hand grip strength (*r* = −.397, *P* < .001), walking speed (*r* = −.161, *P* = .002) and RSMMI (*r* = −.218, *P* < .001; Figure 5D‐F). The association between BMI and muscle parameter showed significant difference in RSMMI (*r* = .465, *P* < .001) and walking speed (*r* = −.059, *P* = .021; Figure 5G,I). The association between BMI and hand grip did not show significance (Figure 5H). After adjustment for demographic and socioeconomic status (Table 5), the multivariate logistic regression model showed that fat mass was an independent risk factor for sarcopenia (OR = 1.064, 95% CI [1.017, 1.113], *P* = .007). In contrast, BMI was independently protective against sarcopenia (OR = 0.423, 95% CI [0.204, 0.887], *P* = .021], especially for overweight participants (OR = 0.121, 95% CI [0.047, 0.307], *P* < .001). CC was also protective against sarcopenia (OR = 0.780, 95% CI [0.733, 0.830], *P* < .001). These results suggest that the body distribution of fat interacts with the loss of muscle in sarcopenia.

### 3.8 Risk factor cut‐off points for sarcopenia

**FIGURE 6:**
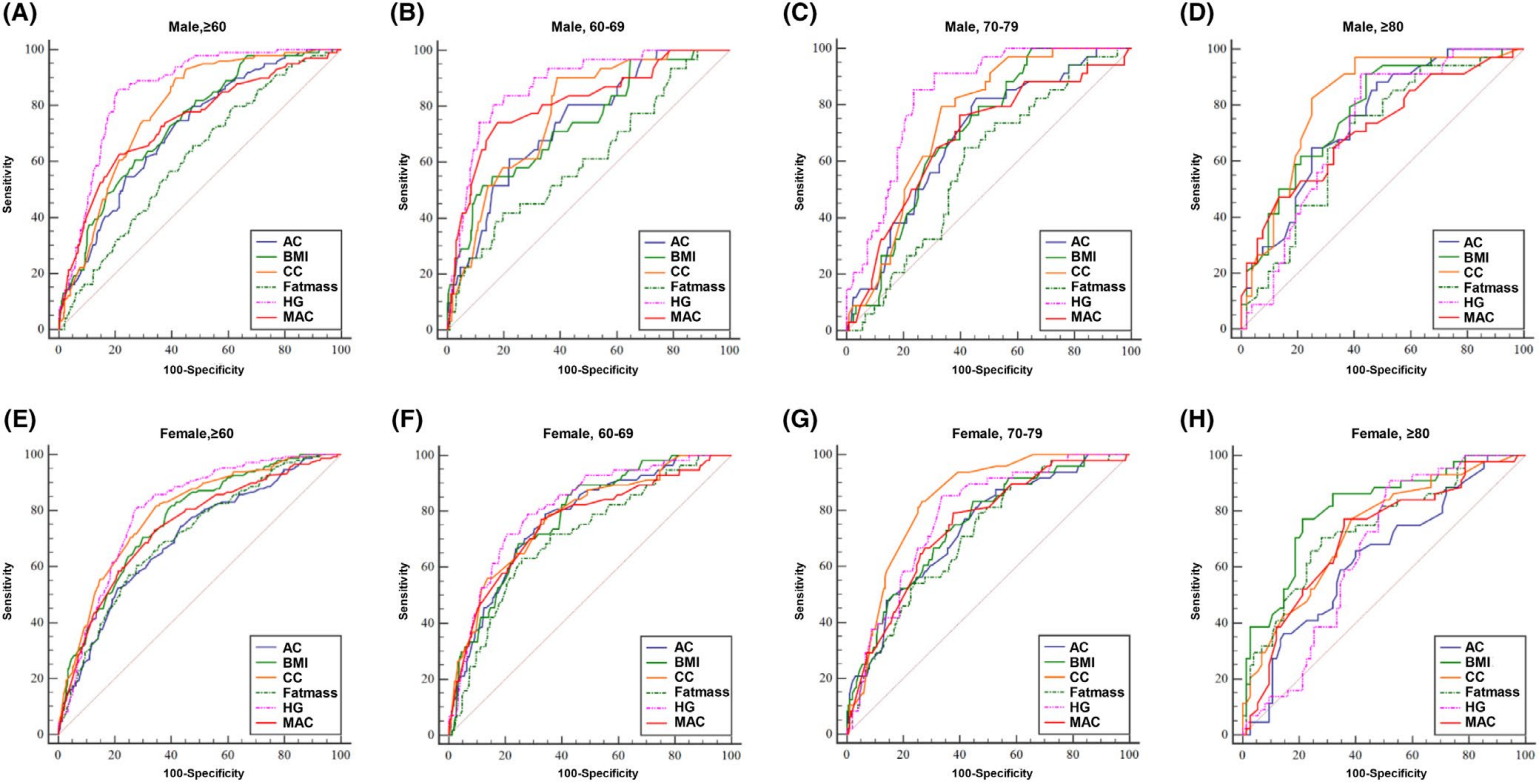
Receiver operating characteristic (ROC) curve analyses of various anthropometric indicators for sarcopenia, based on the AWGS definition. ROC curves are shown for (A) males ≥ 60 years, (B) males 60‐69 years, (C) males 70‐79 years, (D) males ≥ 80 years, (E) females ≥ 60 years, (F) females 60‐69 years, (G) females 70‐79 years, (H) females ≥ 80 years. AC, abdominal circumference; AWGS, Asian Working Group for Sarcopenia; BMI, body mass index; CC, calf circumference; HG, hand grip; MAC, mid‐upper arm circumference

For improved diagnosis of sarcopenia, we aimed to find easily measurable anthropometric variables that could be used to replace muscle mass measurements, such as RSMMI, which are still inconvenient to obtain in most clinics today. From our ROC analysis, the optimal cut‐off value for age, significantly defined as area under the curve (AUC [area under the curve] = 0.699, 95% CI [0.675‐0.722], *P* < .001, Figure S1 ), was actually 71 years (sensitivity, 59.7%; specificity, 73.3%). We compared grip strength, fat mass, BMI, AC, MAC and CC to confirm gender‐ and age group–specific cut‐off points (Figure 6 and Table 6).

**TABLE 6:** ROC analysis to detect CC, MAC, AC, HG, BMI and FM cut‐off points for sarcopenia in the elderly population according to AWGS criterion

	Youden Index		Cut‐offs		Sensitivity		Specificity		AUC (95%CI)		*P* value	
	Male	Female	Male	Female	Male	Female	Male	Female	Male	Female	Male	Female
AC (cm)												
60‐69y	0.393	0.448	≤84.4	≤84.8	61.29	78.95	77.99	65.89	0.741 (0.692‐0.786)	0.772 (0.735‐0.806)	<.001	<.001
70‐79 years	0.368	0.356	≤91.0	≤89	82.35	83.33	54.47	52.26	0.689 (0.610‐0.760)	0.732 (0.673‐0.787)	<.001	<.001
≥80 years	0.402	0.259	≤93.4	≤89.5	88.24	65.91	51.92	60.00	0.746 (0.640‐0.834)	0.635 (0.541‐0.721)	<.001	.01
≥60 years	0.328	0.313	≤90.0	≤86.6	74.75	52.35	58.01	78.91	0.712 (0.674‐0.748)	0.700 (0.670‐0.729)	<.001	<.001
CC (cm)												
60‐69 years	0.513	0.435	≤35.4	≤33.5	90.32	78.95	61.01	64.52	0.775 (0.728‐0.818)	0.780 (0.744‐0.814)	<.001	<.001
70‐79 years	0.461	0.567	≤34.0	≤32.5	79.41	83.33	66.67	73.37	0.748 (0.672‐0.813)	0.833 (0.781‐0.877)	<.001	<.001
≥80 years	0.574	0.386	≤34.3	≤33.0	82.35	77.27	75.00	61.33	0.815 (0.717‐0.891)	0.732 (0.643‐0.809)	<.001	<.001
≥60 years	0.485	0.472	≤35.4	≤33.0	89.9	81.88	58.62	65.31	0.783 (0.748‐0.816)	0.791 (0.764‐0.817)	<.001	<.001
MAC (cm)												
60‐69 years	0.563	0.444	≤26.0	≤26.8	74.19	77.19	82.08	67.25	0.812 (0.767‐0.852)	0.759 (0.722‐0.794)	<.001	<.001
70‐79 years	0.366	0.415	≤27.6	≤26.5	76.47	79.17	60.16	62.31	0.683 (0.604‐0.755)	0.743 (0.683‐0.796)	<.001	<.001
≥80 years	0.336	0.413	≤25.0	≤27.1	47.06	77.27	86.54	64.00	0.715 (0.607‐0.807)	0.707 (0.617‐0.787)	<.001	<.001
≥60 years	0.411	0.390	≤26.0	≤26.8	62.63	73.15	78.50	65.82	0.739 (0.702‐0.774)	0.740 (0.710‐0.768)	<.001	<.001
BMI (kg/m^2^)												
60‐69 years	0.390	0.439	≤21.3	≤24.8	51.61	87.72	87.42	56.14	0.738 (0.689‐0.784)	0.777 (0.741‐0.811)	<.001	<.001
70‐79 years	0.350	0.386	≤26.8	≤25.2	100.00	83.33	34.96	55.28	0.704 (0.626‐0.774)	0.747 (0.688‐0.800)	<.001	<.001
≥80 years	0.470	0.559	≤25.0	≤23.6	91.18	77.27	55.77	78.67	0.774 (0.671‐0.857)	0.812 (0.730‐0.878)	<.001	<.001
≥60 years	0.336	0.417	≤23.3	≤24.6	60.61	81.88	73.02	59.85	0.736 (0.699‐0.771)	0.765 (0.737‐0.792)	<.001	<.001
Fat mass (kg)												
60‐69 years	0.221	0.372	≤14.1	≤18.8	41.94	63.16	80.19	74.07	0.612 (0.558‐0.663)	0.709 (0.670‐0.746)	.045	<.001
70‐79 years	0.232	0.324	≤19.7	≤23.2	64.71	79.17	58.54	53.27	0.588 (0.506‐0.665)	0.708 (0.647‐0.764)	.087	<.001
≥80 years	0.361	0.425	≤20.7	≤20.1	76.47	70.45	59.62	72.00	0.689 (0.581‐0.785)	0.741 (0.653‐0.817)	.001	.01
≥60 years	0.180	0.326	≤19.7	≤19.2	65.66	60.40	52.33	72.17	0.613 (0.572‐0.652)	0.707 (0.677‐0.736)	<.001	<.001
Hand grip (kg)												
60‐69 years	0.643	0.513	≤26.8	≤17.8	80.65	71.93	83.65	79.34	0.875 (0.835‐0.908)	0.810 (0.776‐0.842)	<.001	<.001
70‐79 years	0.617	0.518	≤25.8	≤18.1	85.29	85.42	76.42	66.33	0.837 (0.769‐0.891)	0.780 (0.723‐0.830)	<.001	<.001
≥80 years	0.489	0.389	≤25.9	≤18.5	91.18	90.91	57.69	48.00	0.718 (0.611‐0.810)	0.656 (0.563‐0.741)	<.001	.002
≥60 years	0.648	0.532	≤26.8	≤18.0	85.86	80.54	78.90	72.68	0.856 (0.825‐0.883)	0.801 (0.774‐0.826)	<.001	<.001

Abbreviations: AC, abdominal circumference; CC, calf circumference; MAC, mid‐arm circumference.

For all elderly men above 60 years, the best cut‐off points for grip strength, fat mass, BMI, AC, CC and MAC were 26.8 kg, 19.7 kg, 23.3 kg/m^2^, 90.0 cm, 35.4 cm and 26.0 cm, respectively. We found that the AUCs for AC, CC, MAC, BMI and grip strength were all significant for elderly men (*P* < .001). For 60‐69 years elderly men, the optimal predictors were grip strength, MAC and CC, as confirmed by AUC > 0.75 and Youden index >0.5 (Table 6).

For all elderly women above 60 years, the best cut‐off points for grip strength, fat mass, BMI, AC, CC and MAC were 18.0 kg, 19.2 kg, 24.6 kg/m^2^, 86.6 cm, 33.0 cm and 26.8 cm, respectively. We found that the AUCs for AC, fat mass, grip strength and MAC were all significant for 60‐69 years and 70‐79 years elderly women (*P* < .001). The optimal predictors were grip strength for 60‐69 years women, grip strength and CC for 70‐79 years women, and BMI for elderly women above 80 years, as confirmed by AUC > 0.75 and Youden index > 0.5 (Table 6).

Overall, the best predictor(s) of sarcopenia from the ROC analysis, statistically defined as the best compromise between sensitivity and specificity, were grip strength and CC for men (sensitivity, 85.9%; specificity, 78.9%; and sensitivity, 89.9%; specificity, 58.6%), and grip strength (sensitivity, 80.5%; specificity, 72.7%), BMI (sensitivity, 81.9%; specificity, 59.9%) and CC for women (sensitivity, 81.9%; specificity, 65.3%). This is surprising and interesting because previous EWGSOP criteria had always used SMI, grip strength and walking speed for both men and women. Our ROC analysis suggests that different diagnostic variables should be used for men vs women, and perhaps even different age groups of men and women.

### 3.9 PUMCHS index for predicting sarcopenia in men and women

Based on the above comprehensive analysis for sarcopenia, gender appeared to play an important role in diagnosis and pathogenesis. Hence, the predictive model was calculated separately for men and women. In the univariate analysis, the following parameters were identified as associated with sarcopenia for men: age, nutrition status, BMI, AC, MAC, CC, fat‐free mass, fat mass and grip strength (*P* < .001), marital status (*P* = .001), bean frequency (*P* = .001), meat frequency (*P* = .030), meat intake (*P* = .011) and bean intake (*P* = .030). For women, age, nutrition status, marital status, BMI, AC, MAC, CC and grip strength (all *P* < .001), living situation (*P* = .019), smoking (*P* = .013), ADL (*P* = .003), appetite (*P* = .009), total protein (*P* = .005), animal protein (*P* = .018), meat frequency (*P* = .001), bean frequency (*P* = .012), vegetable intake (*P* = .003) and nut frequency (*P* = .032) were associated with sarcopenia. According to a series of multicollinearity analyses, we eliminated AC (*r* = .740 with BMI, *P* < .001), MAC (*r* = .557 with BMI, *P* < .001) and animal protein (*r* = .793 with total protein, *P* < .001) for women, and eliminated AC (*r* = .744 with BMI, *P* < .001) and MAC (*r* = .556 with BMI, *P* < .001) for men.

In the multivariate analysis using forward conditional stepwise procedures, age (OR = 1.070, 95% CI [1.040‐1.100], *P* < .001), BMI (OR = 0.749, 95% CI [0.692‐0.810], *P* < .001), grip strength (OR = 0.766, 95% CI [0.721‐0.813], *P* < .001) and CC (OR = 0.826, 95% CI [0.757‐0.901], *P* < .001) emerged as independent predictors of sarcopenia in women. For men, age (OR = 1.067, 95% CI [1.030‐1.105], *P* < .001), BMI (OR = 0.772, 95% CI [0.697‐0.856], *P* < .001), CC (OR = 0.860, 95% CI [.777‐0.951], *P* = .003) and grip strength (OR = 0.822, 95% CI [0.784‐0.863], *P* < .001) emerged as the independent predictors of sarcopenia. Using these independent predictors, we derived a new model for predicting sarcopenia, named the PUMCHS (Peking Union Medical College Hospital Sarcopenia) index:

$$\text{PUMCHS index}=\frac{1}{1+e^{‐\left(a+b\beta +c\gamma +d\delta +e\varepsilon \right)}}$$

where *β* is the BMI (kg/m^2^), *γ* the grip strength (kg), *δ* the CC (cm), *ε* the age (years), and *a*, *b*, *c*, *d* and *e* are the respective coefficients generated by the model. For women, *a* = 11.554, *b* = −0.267, *c* = −0.29, *d* = −0.191, *e* = 0.067. For men, *a* = 10.229, *b* = −0.258, *c* = −0.195, *d* = −0.151, *e* = 0.065.

The PUMCHS index was evaluated by the ROC curves for the entire population, and the cut‐off points for the index were 0.172 for men and 0.186 for women. The AUC was 0.905 (95% CI [0.885‐0.923], *P* < .001) for women and 0.920 (95% CI [0.895‐0.940], *P* < .001) for men. These values indicate the PUMCHS index had very high predictive power for both men and women.

In addition, comprehensive analysis of the key predictors in the PUMCHS index, BMI, CC and grip strength revealed that these predictive variables agglomerated the differing and complex influences that nutrition exerted on men and women. For example, BMI was significantly correlated with meat intake, meat frequency, milk frequency, poultry intake, vegetable protein, bean intake, nut frequency, fruit intake, grain intake, salt intake and oil intake (all *P* < .05; Table 7 and Figure 4), to very different degrees for male vs female. In contrast, grip strength was significantly correlated with total protein, animal protein, vegetable protein, meat frequency, meat intake, fish intake, poultry intake, bean frequency, bean intake, vegetable intake, nut frequency, nut intake, fruit intake, grain intake and salt intake (all *P* < .05; Table 7 and Figure 4), to differing degrees for men vs women. CC was significantly correlated with vegetable protein, meat frequency, milk frequency, bean frequency, grain intake, milk intake, fruit intake, vegetable intake, meat intake, bean intake, salt intake and oil intake (all *P* < .05, Table 7), to very different degrees for men vs women. Combined with the daily distribution of protein intake and age, the PUMCHS index is a simple yet powerful model to predict the risk for sarcopenia, based on the differential effects of nutrition on elderly men and women.

**TABLE 7:** Associations between dietary parameters versus BMI and handgrip strength in elderly (≥60 years) subjects

	BMI (kg/m^2^)				Handgrip strength (kg)				Calf circumstance (cm)			
	Male		Female		Male		Female		Male		Female	
	*R* value	*P* value	*R* value	*P* value	*R* value	*P* value	*R* value	*P* value	*R* value	*P* value	*R* value	*P* value
Total protein (g/day)	.025	.55	.006	.85	.015	.71	.114	<.001	.002	.97	.014	.68
Animal protein (g/day)	−.019	.64	−.019	.57	−.015	.72	.112	.001	−.07	.091	−.05	.12
Vegetable protein (g/day)	.097	.019	.021	.53	.056	.18	.064	.051	.094	.023	.070	.033
Protein distribution (g/day)	−.056	.17	.037	.26	.083	.043	−.014	.66	−.039	.34	.021	.53
Fish frequency	−.029	.48	.05	.13	.041	.33	−.038	.25	.030	.47	.051	.12
Poultry frequency	−.06	.15	.014	.67	.007	.86	−.048	.14	−.042	.31	.030	.36
Meat frequency	−.136	.001	.021	.52	−.104	.012	−.151	<.001	−.225	<.001	−.109	.001
Milk frequency	.072	.082	.138	<.001	.078	.059	−.063	.056	.084	.041	.067	.039
Bean frequency	−.082	.047	−.015	.66	−.111	.007	−.143	<.001	−.125	.002	−.133	<.001
Nut frequency	.015	.72	.071	.031	−.006	.88	−.151	<.001	−.015	.72	−.019	.57
Caffeine drinking	−.033	.42	.004	.9	.096	.02	.120	<.001	−.027	.51	.044	.18
25‐hydroxyvitamin D	.071	.16	.117	.007	.249	<.001	−.055	.21	.156	.002	.025	.57
Grain intake (g/day)	−.07	.088	−.076	.02	−.095	.021	−.045	.17	−.086	.037	−.056	.09
Milk intake (mL/time)	.063	.13	.084	.01	.093	.024	−.002	.95	.082	.048	.052	.11
Fish intake (g/day)	−.047	.25	−.039	.24	−.018	.66	−.155	<.001	−.043	.30	−.068	.038
Meat intake (g/day)	−.021	.62	−.072	.027	−.09	.029	−.100	.002	−.084	.041	−.064	.049
Poultry intake (g/day)	−.104	.011	−.051	.12	−.021	.61	−.117	<.001	−.062	.13	−.017	.61
Vegetable intake (g/day)	−.039	.34	.017	.6	−.126	.002	−.181	<.001	−.09	.029	−.081	.014
Fruit intake (g/day)	.026	.54	.119	<.001	−.082	.046	−.074	.023	.059	.16	.098	.003
Bean intake (g/day)	−.121	.003	−.062	.059	−.021	.62	−.069	.035	−.156	<.001	−.096	.003
Nut intake (g/day)	−.025	.54	.016	.62	−.005	.9	−.133	<.001	−.039	.34	−.012	.72
Salt intake (g/day)	.032	.43	.115	<.001	.093	.023	.088	.007	.143	<.001	.141	<.001
Oil intake (g/day)	.124	.003	.088	.007	.038	.36	.001	.99	.119	.004	.113	.001

## 4 DISCUSSION

Among the geriatric participants from the BELFRAIL study, more than half had both reduced grip strength and limited walking speed, but with normal muscle mass.^8^ Longitudinal studies have found that low grip strength represents a predictor of functional disability and mortality and that grip strength and/or physical performance capacity are better predictors of clinical outcome than muscle mass.^8^ For this reason, more research was necessary to determine the reference values and appropriate diagnostic methods for muscle strength and function. In our study, the prevalence of probable sarcopenia (defined by low muscle strength, 25.9%) was higher than pre‐sarcopenia (defined by low muscle mass, 10.1%) in elderly persons, and an ageing trend was obvious in probable sarcopenia (Table 2). Risk prediction studies do not necessarily include causes or targets of intervention, but may include biomarkers of causes that are cheap and easy to measure, which is what we focused on. Our ROC analysis of various anthropometric indicators of muscle mass and function showed that hand grip strength was the best predictor of sarcopenia in both men and women, but BMI was still a useful predictor of sarcopenia for both sexes as well. Accordingly, both BMI and grip strength were included in the PUMCHS index. Age also emerged as one of the critical independent predictors of sarcopenia in our multivariate analysis, especially for the PUMCHS index. After surveying a variety of nutritional, lifestyle and anthropometric variables, we uncovered several biomarkers that we incorporated into an index for more convenient prediction of sarcopenia risk in the clinic. Although muscle mass was a critical factor for diagnosing sarcopenia, the verified PUMCHS index (AUC 0.905 for men and 0.920 for women, *P* < .001) might be a more convenient, cost‐saving and time‐saving tool to identify high‐risk populations of sarcopenia, for both urban and rural clinics.

Age was a risk factor for all stages of sarcopenia. From our ROC analysis, the optimal cut‐off value for age was 71 years (sensitivity, 59.7%; specificity, 73.3%). This is important because EWGSOP2 (2019) provided a definite cut‐off point for age,^1^ but EWGSOP (2010) had not, leading to some confusion in the field. Our PUMCHS index clarifies the relative diagnostic value, for both men and women.

The wealth of gender‐specific associations uncovered by our study supports a more complete revision of male versus female sarcopenia diagnosis. In our study, men suffered more frequently from sarcopenia in almost all stages. Gallagher et al^21^ also found that the amount of muscle mass lost in men was approximately double than that in women at the same age. Although at every age the total muscle mass of men is greater than that of women, men experience a greater loss of skeletal muscle mass,^19^ as further confirmed by our study. Others have postulated that age‐related androgen deficiency could be a cause for this phenomenon, since total testosterone levels decrease by ~1% and bioactive free testosterone levels drop by ~2% per year in men after 30 years of age,^22^ a phenomenon that our study also confirmed in Asian Chinese participants. Since there were technical difficulties with measuring the low levels of oestrogen in elderly women, due to the influence of menopause, and elderly men also show very low levels of oestrogen, we necessarily had to focus on the effects of androgens in this study.

Our findings on gender, testosterone and vitamin D led us to a deeper examination of the role of nutrition. Nutrition is known to play an important role in the development of sarcopenia, as food and special nutrient intakes decline gradually throughout adulthood. A number of studies have shown that protein intake is a key factor for optimal muscle and bone health in older adults.^23^ However, determination of the optimal quantity, frequency and subtype of protein intake to preserve muscle mass and function has even greater practical value. Our present findings showed significant associations between sarcopenia and protein intake, especially meat and bean intake, but to varying degrees among men vs women. Meat intake less than once per week doubled the risk for sarcopenia, and sarcopenia risk further doubled with zero meat intake, which is a common practice of vegetarians in China. Interestingly, our data further indicated that sufficient protein intake (1.2 g/kg body weight/day) concentrated in one meal per day reduced the risk of sarcopenia by over threefold, likely through stimulating insulin/IGF‐PI3K‐mTOR signalling and increasing protein synthesis to conserve muscle mass in older adults.^24^ Surprisingly, our data also showed that bean intake has a protective effect on preserving muscle and preventing sarcopenia in elderly people. This new finding adds to a growing list of health benefits that has been ascribed to bean consumption in nutritional and epidemiological studies. Furthermore, we can direct patients increasing the frequency and intake of meat and bean and changing the model of protein intake. Furthermore, the dietary patterns between gender should be differentiated for maintaining muscle mass and function; for example, the elderly women can increase the nut snacks, which can improve the quality of life for women. Further studies would be needed to verify the effects and cross‐interactions of specific food nutrients and metabolites on muscle mass and function.

Among all the predictors of sarcopenia in our study, hand grip strength was consistently the best diagnostic variable for predicting sarcopenia (AUC = 0.856 in males; 0.801 in females), which was consistent with the EWGSOP2 definition of probable sarcopenia. Hand grip strength correlates moderately with strength in the arms and leg muscles, and frequently serves as a proxy for global muscle strength.^25^ In our study of elderly persons in Asian Chinese, the cut‐off points of <26.8 kg in men and <18.0 kg in women best predicted the risk of sarcopenia. Moreover, hand grip strength emerged from all other diagnostic variables as one of the critical independent predictors of sarcopenic risk in our PUMCHS index, for both men and women. Retrospective analysis revealed that hand grip strength encapsulated the complex influences of nutrition and incorporated correlations with intake of total protein, meat, fish, poultry, bean, vegetable, nut, fruit, grain, salt and fat percentage (all *P* < .05), but to differing degrees for men vs. women (Table 7).

However, as this was a cross‐sectional study, the exact causal relationships between muscle mass/function and the various variables could not be precisely determined. While our analyses clearly indicate that several extrinsic variables, such as dietary factors related to the intake of protein, fat, sterols, vitamin D (cholecalciferol) and caffeine, can differentially influence the risk for sarcopenia in men and women, outcome‐based clinical trials and human cell experiments will still be needed to confirm the effects of these metabolic perturbations and dietary interventions. It should also be noted that the study subjects excluded from the analyses for various technical reasons were generally older with worse health conditions, which could mean we underestimated the prevalence of sarcopenia in Asian Chinese as a result.

Nevertheless, among the confusing array of muscle parameters used for clinical definitions of sarcopenia, our epidemiological data analyses suggest that gender, sterol metabolism, BMI, grip strength, calf circumstance and age stand out as the most important predictors to consider for improving the accurate standardization of sarcopenia assessment for future studies.

## CONFLICT OF INTEREST

All authors declare that they have no conflict of interest.

## Supporting information

Fig S1

## Data Availability

The data that support the findings of this study are available on request from the corresponding author. The data are not publicly available due to privacy or ethical restrictions.
