# Supplementary figures and images for: Sterol metabolism and protein metabolism are differentially correlated with sarcopenia in Asian Chinese men and women
Source: Cell Prolif. 2021 Feb 20;54(4):e12989. doi: 10.1111/cpr.12989 (PMC8016649; doi:10.1111/cpr.12989)

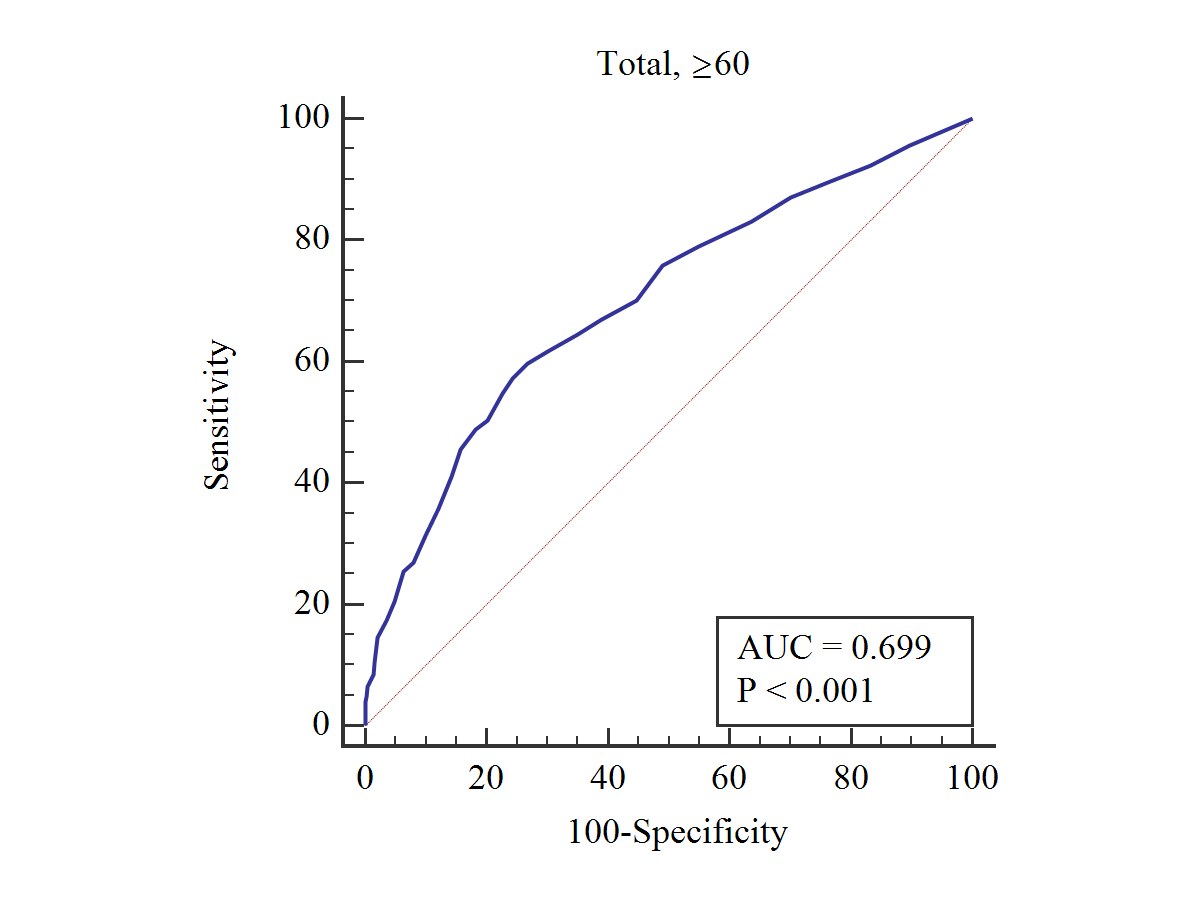

Supplement: Supplementary file 1 — Fig S1 [file CPR-54-e12989-s001.tif]
